# Glioblastoma‐educated mesenchymal stem‐like cells promote glioblastoma infiltration via extracellular matrix remodelling in the tumour microenvironment

**DOI:** 10.1002/ctm2.997

**Published:** 2022-07-31

**Authors:** Seung‐Mo Kim, Eun‐Jung Lim, Ki‐Chun Yoo, Yi Zhao, Jae‐Hyeok Kang, Eun‐Ji Lim, Incheol Shin, Seok‐Gu Kang, Han Woong Lim, Su‐Jae Lee

**Affiliations:** ^1^ Department of Life Science Research Institute for Natural Sciences Hanyang University Seoul Korea; ^2^ Memorial Sloan Kettering, Cancer Center New York New York USA; ^3^ Department of Lymphoma and Myeloma Division of Cancer Medicine Center for Cancer Immunology Research The University of Texas MD Anderson Cancer Center Houston Texas USA; ^4^ Department of Neurosurgery Brain Tumor Center, Severance Hospital Yonsei University College of Medicine Seoul Korea; ^5^ Department of Ophthalmology Hanyang University Hospital Hanyang University College of Medicine Seoul Korea; ^6^ Fibrosis and Cancer Targeting Biotechnology FNCT Biotech Seoul Korea

**Keywords:** cluster of differentiation 40 ligand, extracellular matrix remodelling, glioblastoma, lysyl oxidase, mesenchymal stem‐like cells

## Abstract

**Background:**

The biological function of mesenchymal stem‐like cells (MSLCs), a type of stromal cells, in the regulation of the tumour microenvironment is unclear. Here, we investigated the molecular mechanisms underlying extracellular matrix (ECM) remodelling and crosstalk between MSLCs and glioblastomas (GBMs) in tumour progression.

**Methods:**

In vitro and in vivo co‐culture systems were used to analyze ECM remodelling and GBM infiltration. In addition, clinical databases, samples from patients with GBM and a xenografted mouse model of GBM were used.

**Results:**

Previous studies have shown that the survival of patients with GBM from whom MSLCs could be isolated is substantially shorter than that of patients from whom MSLCs could not be isolated. Therefore, we determined the correlation between changes in ECM‐related gene expression in MSLC‐isolatable patients with that in MSLC non‐isolatable patients using gene set enrichment analysis (GSEA). We found that lysyl oxidase (LOX) and COL1A1 expressions increased in MSLCs via GBM‐derived clusters of differentiation 40 ligand (CD40L). Mechanistically, MSLCs are reprogrammed by the CD40L/CD40/NFκB2 signalling axis to build a tumour infiltrative microenvironment involving collagen crosslinking. Importantly, blocking of CD40L by a neutralizing antibody‐suppressed LOX expression and ECM remodelling, decreasing GBM infiltration in mouse xenograft models. Clinically, high expression of CD40L, clusters of differentiation 40 (CD40) and LOX correlated with poor survival in patients with glioma. This indicated that GBM‐educated MSLCs promote GBM infiltration via ECM remodelling in the tumour microenvironment.

**Conclusion:**

Our findings provide mechanistic insights into the pro‐infiltrative tumour microenvironment produced by GBM‐educated MSLCs and highlight a potential therapeutic target that can be used for suppressing GBM infiltration.

## Graphical abstract

- In GBM patients, the survival rate of MSLC isolatable patients is lower than MSLC non‐isolatable patients, and ECM remodeling is more active.
- GBM‐secreted CD40L educates MSLCs, and educated‐MSLCs promote ECM remodeling for GBM infiltration via CD40/NFκB2/LOX signaling axis.
- When MSLCs are present in the GBM microenvironment, which could inhibition of GBM infiltration by treatment with CD40L blockade.

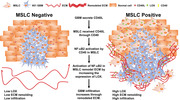

## 1 INTRODUCTION

Glioblastoma (GBM), also known as grade IV glioma, is the most common and malignant type of primary brain cancer. Despite the availability of advanced multimodal therapy for patients with GBM, which involves surgery, radiotherapy and chemotherapy, median survival has been reported to be less than 15 months.^1^, ^2^ Surgical resection is the first line of treatment applied to most patients with brain tumours, but in the case of malignant gliomas, completely removing the tumour due to its high diffuseness is difficult, resulting in a high probability of recurrence and poor prognosis.^3^, ^4^ Moreover, a growing body of evidence has shown that anti‐cancer drugs are not effective in improving the overall survival of patients with GBM due to various factors exerting intrinsic or extrinsic effects of GBM microenvironment such as tumour heterogeneity, metabolic change, immune regulation and the blood–brain barrier.^2^, ^5^, ^6^, ^7^, ^8^, ^9^ This indicates that new strategies for treating GBM are absolutely necessary.

Most approaches for treating cancer are focused on the intrinsic properties of tumour cells, but there has been increasing interest on the tumour microenvironment (TME); for instance, the success of immune checkpoint blockades (ICBs). The TME consists of not only tumour cells, but also non‐cancerous cells, such as endothelial cells, pericytes, fibroblasts, immune cells and inflammatory cells, and non‐cellular components such as growth factors, cytokines, chemokines and extracellular matrix (ECM) that surround the tumour.^10^, ^11^ The crosstalk between tumour cells and TME is similar to the relationship between the ‘seed’ and ‘soil’, and cancer progression is closely related to the TME. Emerging evidence suggests that cancer cells can invade the surrounding normal tissue and metastasize to secondary organs via crosstalk with stromal cells.^12^, ^13^

Mesenchymal stem cells (MSCs), a type of multipotent stromal stem cells, can differentiate into chondrocytes, osteoblasts and adipocytes. MSCs have been identified to be the stromal component in many types of cancer, including GBM. Furthermore, the MSCs in TME migrate toward tumour sites and possess both tumour‐promoting and tumour‐suppressing abilities depending on their origin and tumour type.^14^, ^15^, ^16^ Consistent with this, studies have demonstrated that tumour progression in glioma is driven by an increase in proliferation and migration, and MSCs increase proliferation and maintain stemness in glioma via the IL‐6/gp130/STAT3 pathway.^17^, ^18^ We have also reported the presence of mesenchymal stem‐like cells (MSLCs) in GBM^19^ and have shown that they affect GBM progression via the activation of the C5a/P38/ZEB1 pathway.^20^ However, how pro‐tumoral MSCs or MSLCs are controlled by GBM cells, and how they regulate the latter's invasiveness is not understood.

Cluster of differentiation 40 ligand (CD40L), also known as CD154, is a member of the tumour necrosis factor family of cell transmembrane proteins and mainly expressed in activated T cells, endothelial cells and platelets. It regulates B‐cell maturation and function by engaging clusters of differentiation 40 (CD40) on the B‐cell surface.^21^ PD‐L1 and CTLA1 are the main immune checkpoint proteins. Several studies have suggested that CD40L/CD40 are important targets for the next generation of immune checkpoint proteins in cancer therapy.^22^ Furthermore, a high expression of CD40L in cancer is related to an increase in proliferation and poor prognosis.^23^, ^24^ Although the importance of CD40L in the treatment of some cancer types is well known, the molecular mechanism of its action and its functions in GBM remain unknown.

Remodelling of ECM molecules, which are essential components of the TME, is the main determinant controlling the development, survival and invasion of cancer cells via biochemical and biomechanical cues.^25^, ^26^ Furthermore, accumulation and crosslinking of collagen are critically involved in biological activities and cell signalling in cancer. Lysyl oxidase (LOX) is a secreted copper‐dependent amine oxidase that catalyzes the covalent crosslinking of collagen and elastin via oxidation in the TME, and increases matrix deposition, structural stability and tensile strength.^27^, ^28^ Although, accumulating evidence has shown that increased crosslinking of collagens by LOX is closely associated with cancer progression.^29^ In contrast, some studies have suggested that LOX‐propeptide acts as a tumour suppressor.^30^ As such, whether LOX in the TME induces tumour progression or suppression still remains obscure.

In this study, we investigated the decrease in patient survival rate occurring when MSLCs are present in the GBM microenvironment due to mechanical remodelling by CD40L‐reprogrammed MSLC. GBM cells secreted CD40L around the tumour and reprogrammed MSLCs through CD40. The reprogrammed MSLCs secrete LOX and promote the invasive properties of GBM cells by ECM remodelling. Our results indicate that LOX and CD40L may be developed as rationale targets for controlling GBM invasion in the GBM microenvironment.

## 2 MATERIALS AND METHODS

### 2.1 Culturing GBM cells and MSLCs

Patient‐derived X01 (mesenchymal type) and X02 GBM cells established from an acutely excised human GBM biopsy were generously provided by Dr. Akio Soeda (Department of Neurological Surgery, University of Virginia, Charlottesville, VA, USA).^31^ GSC‐11 GBM cells (proneural type) were kindly provided by Frederick F. Lang's laboratory (The University of Texas MD Anderson Cancer Center, Houston, TX, USA).^32^ All GBM cells were cultured in serum‐free DMEM‐F12 (Invitrogen, Seoul, Korea) + bFGF (Sigma‐Aldrich, Seoul, Korea) + EGF (20 ng/ml; Sigma‐Aldrich, Korea) supplemented with B27 (Invitrogen, Seoul, Korea) and 1% penicillin/streptomycin and an antibiotic‐antimycotic solution (Gibco, Seoul, Korea). MSLCs 09‐03 were isolated from human GBM biopsies.^33^ U87, U251 and U373 GBM cells were cultured in Dulbecco's Modified Eagle Medium (DMEM) and high glucose (Gibco, Thermo Fisher, Seoul, Korea) supplemented with 10% fetal bovine serum (FBS) and 1% penicillin/streptomycin and an antibiotic‐antimycotic solution. MSLCs 09‐03 were cultured in Eagle's Minimal Essential Medium (MEMα) (Corning Inc., NY, USA) supplemented with 10% FBS and 1% penicillin/streptomycin and an antibiotic‐antimycotic solution. Astrocytes were purchased from Lonza (Lonza, Basel, Switzerland). Astrocytes were cultured using astrocyte basal medium (ABM) with supplements (astrocyte growth medium [AGM]; FBS, L‐glutamine, gentamicin sulfate‐amphotericin, ascorbic acid, HEGF, insulin), as recommended by the manufacturer. All cell lines were negative for *Mycoplasma* contamination. The conditioned media (CM) was harvested from each cells cultured media, filtered through a 0.22‐μm filter and stored at −80°C.

### 2.2 Antibodies and reagents

Antibodies to CD40L, CD40, IκBα, NF‐κB(p50), NF‐κB(p52), lamin B1, β‐tubulin, STAT3, Akt1 and ERK 1/2 were purchased from Santa Cruz (Santa Cruz Biotechnology, Dallas, TX, USA). LOX and collagen I, CD105(M), CD105(R), CD44, IBA‐1, CD45 and ZEB1 antibodies were purchased from Abcam (Cambridge, UK). Antibodies to phospho‐Stat3 (Tyr705), phospho‐p38 MAPK, p38, phospho‐Akt (Ser473), phospho‐Src (Tyr527), SRC, JNK, phospho‐SAPK/JNK (Thr183/Tyr185) and p44/42 MAPK (Erk1/2) were purchased from Cell Signaling (Beverly, MA, USA). Collagen I (high concentration, rat tail, 100 mg) was purchased from Corning (NY, USA). Anti‐rabbit Ig‐HRP, anti‐goat IgG‐HRP and anti‐mouse IgG‐HRP were purchased from GeneTex (Irvine, CA, USA). Anti‐mouse Alexa Fluor 488, anti‐rabbit Alexa Fluor 488, anti‐mouse Alexa Fluor 546 and anti‐rabbit Alexa Fluor 546 were purchased from Invitrogen (Carlsbad, CA, USA). Recombinant human CD40L (rhCD40L) was purchased from R&D system (Minneapolis, MN, USA) and recombinant human LOX (rhLOX) active protein was purchased from Mybiosource (MBS2097305, San Diego, CA, USA). β‐Aminopropionitrile (BAPN) was purchased from Sigma Aldrich (A3134, St. Louis, MO, USA).

### 2.3 Co‐culture of MSLCs and GBM cells

GBM cells (2 × 10^5^) were seeded in a six‐well plate (diameter: 35 mm, depth: 17.5 mm), and MSLCs (2 × 10^5^) were seeded in the upper transwell chamber (3412, Corning, ME, USA; pore size of 0.4 μm). Cell changes due to crosstalk were analyzed after co‐culture for 3 days.

### 2.4 Collagen invasion assays

Collagen concentration‐dependent GBM cell invasion was analyzed in transwells (3422, Corning; pore size 0.8 μm) precoated with 3, 6 or 9 mg/ml rat tail collagen type 1 (354249, Corning) for invasion. Collagen‐coated transwells incubated with MSLCs were coated with collagen at a concentration of 3 or 6 mg/ml. Collagen‐coated transwells were incubated for 3 days in a 24‐well plate seeded with MSLCs (5 × 10^4^) or in a 24‐well plate containing concentration‐dependent diluted rhLOX in culture media. The GBM cells (4 × 10^4^) were seeded in the upper transwell chamber and incubated for 72 h. The GBM cells that invaded into the lower surface of the transwell membrane were then stained using a Diff Quick kit (Fisher, Pittsburgh, PA, USA). The number of invaded cells were counted in three microscopic images per well.

### 2.5 Enzyme‐linked immunosorbent assay (ELISA) and LOX activity assay

CM collected from each cell culture medium and the level of secreted LOX (MBS039099, MyBioSource, San Diego, CA, USA), Collagen1A1 (MBS763786, MyBioSource, San Diego, CA, USA) and CD40L (DCDL40, R&D system, MN, USA) were measured using ELISA according to the manufacturer's instructions. The activity of LOX protein was measured using a Lysyl Oxidase Activity Assay kit (ab112139, Abcam, Cambridge, UK) in recombinant active LOX protein or collected CM from each sample according to the manufacturer's instructions. Collected CM was filtered through a 0.22‐μm filter, and stored at −80°C.

### 2.6 ECM remodelled by MSLCs to GBM invasion

For collagen‐based matrix, rat tail collagen type 1 (final concentration 2 mg/ml), matrigel (11% v/v) and reconstitution buffer (26 mM NaHCO_3_, 5 mM NaOH, 20 mM HEPES in serum‐free MEM) were mixed on ice. The prepared collagen‐based matrix was mixed with each cell or recombinant LOX, placed into a Millicell culture plate insert (12 mm diameter, 0.4 μm pore size; Millipore, Billerica, MA, USA), and incubated at 37°C for 1 h, followed by addition of culture media. After 5 days, cells in the collagen‐based matrix were killed by treatment with puromycine for 24 h and then washed for 24 h to withdraw the conditioned ECM prepared by each cell. X01 cells were seeded in the upper matrix of the prepared conditioned ECM and incubated for 3 days to observe cell invasion and matrix crosslinking by polarized light. The prepared collagen‐based matrix was embedded in paraffin and sectioned to a thickness of 4 μm, followed by haematoxylin and eosin (H&E) and Sirius red staining. The schematic model for the experiment is shown in each figure.

### 2.7 3D spheroid invasion assay

A mixture of MSLCs cells and collagen‐based matrix was seeded in a glass bottom confocal dish (SPL, Seoul, Korea). Four hours after seeding, X01 or GSC11 GBM spheroids labelled with green fluorescent protein (GFP) were loaded into collagen‐based matrix and incubated for 24−48 h. Infiltration was quantified by calculating the GFP signalling of whole invaded area (*A*_T_) compared to the GFP signalling of spheroid at the initial time (*A*_0_). Infiltration was quantified using the formula; (*A*_T_ − *A*_0_)/*A*_0_ × 100.

### 2.8 Picrosirius red stain and polarized light microscopy

For Picrosirius red staining, paraffin‐embedded tissue and collagen‐based matrix slides were stained using the Picro Sirius Red Stain Kit (ab150681, Abcam) according to the manufacturer's instructions. Collagen fibre was evaluated on Picrosirius red‐stained collagen‐based matrix and mouse and human tissue slides at 100× using a microscope (BX50, Olympus, Seoul, Korea) with a polarizing filter. For image analysis, photomicrographs were batch processed using Image J software. Initially, photomicrographs were digitized as 8‐bit grayscale images using macros of Image J software. Subsequently, the collagen fibre area was quantified in black and white by adjusting the threshold without damaging the original polarization image.

Every photomicrograph was evaluated on the same 2070 × 1548 pixel image, the collagen fibre area of three images per group was compared with that of the control group, and the collagen fibre area was calculated using the fold change method.

### 2.9 Transfection and establishment of stable cell line

GBM cells (7 × 10^5^ cells) or MSLCs (7 × 10^5^ cells) were transfected with small interfering RNA (si‐RNA) using Microporator‐mini (Digital Bio Technology, Seoul, Korea) according to manufacturer's instructions. Reseeding for co‐culture was performed 48 h after transfection. All si‐RNA were purchased from Genolution Pharmaceuticals, Inc. (Seoul, Korea). The siRNA sequence is shown in Table S1. A CD40‐specific short hairpin RNA (sh‐RNA) was cloned into the lentiviral vector pLKO.1‐puro (Sigma Aldrich). For GFP labelling of GBM cells, GBM cells were cultured in presence of serum condition when transducing GFP expression. For lentiviral production, HEK293T cells were transfected with EFSp‐GFP‐Empty, pLKO.1‐sh‐control or pLKO.1‐sh‐CD40. After 48 h, the produced viral supernatant was filtered through a 0.22‐μm filter and used for transduction.

### 2.10 Western blot analysis

Proteins in cell lysates were separated using SDS‐PAGE and transferred to a nitrocellulose membrane (Amersham, Arlington Heights, IL, USA). The membrane was blocked with 5% nonfat dry milk in Tris‐buffered saline and incubated with primary antibodies overnight at 4°C. The blots were developed with a peroxidase‐conjugated secondary antibody, and the proteins were visualized by enhanced chemiluminescence procedures (Amersham). Quantitative analysis of Western blots was performed using ImageJ software.

### 2.11 Quantitative reverse transcription‐polymerase chain reaction (qRT‐PCR)

RNA isolated from cells using TRIzol reagent was used to synthesize complementary DNA (cDNA). Extracted RNA was quantified using spectrophotometry (NanoDrop; Thermo Scientific, Waltham, MA, USA). The same quantity of 500 ng RNA was used for cDNA synthesis, and cDNA was amplified using universal qPCR kit from KAPA Biosystems (KAPA Biosystems, Wilmington, MA, USA) according to manufacturer's instruction. Real‐time PCR was performed using SensiFAST SYBR No‐ROX (Bioline, Menphis, TN, USA) in Rotor Gene Q (Quiagen, Seoul, Korea). qRT‐PCR was performed following MIQE guidelines.^34^ The primer sequences of qRT‐PCR and the thermocycler conditions are presented in Tables S2 and S4. The expression levels were normalized using β‐actin and GAPDH, and fold‐change values were indicated by the 2^−ΔΔCt^ method.

### 2.12 Chromatin immunoprecipitation (ChIP) assays

Before performing a ChIP assay, cells were crosslinked with 1% formaldehyde. The ChIP assay was performed using the EZ‐ChIP kit (Merck, Darmstadt, Germany) according to the manufacturer's instructions. For immunoprecipitation (IP), anti‐NF‐κB2 antibody and anti‐IgG as negative controls and anti‐RNA polymerase II as a positive control were used. GAPDH was used to verify the accuracy of IP. The transcription factor binding target promoter region was predicted by JASPER (http://jaspar.genereg.net/) and UCSC Genome Browser (https://genome.ucsc.edu/index.html). The primer sequences used in the ChIP‐assay and the thermocycler conditions are presented in Tables S2 and S3.

### 2.13 Cytokine array

A human cytokine array (Proteome Profiler Array Human Cytokine, R&D system, ARY#005B) was used to detect 36 human secretion factors in each cells CM according to the manufacturer's protocol. Cytokine levels were detected using an X‐ray film and quantified using the ImageJ software, considering the positive control.

### 2.14 Immunocytochemistry (ICC)

After fixing the cells with 4% paraformaldehyde, permeabilizing and blocking were performed with phosphate‐buffered saline (PBS) containing 0.2% NP‐40 and 10% FBS. Following fixation, the cells were incubated with primary antibody in blocking buffer at 4°C overnight. After washing the primary antibody, the cells were detected using anti‐mouse Alexa Fluor 488 or anti‐mouse Alexa Fluor 546 conjugated secondary antibody. Cell nuclei were counterstained using 4′,6‐diamidino‐2‐phenylindole (DAPI) (Sigma Aldrich). The stained cells were visualized under a Nikon C2 confocal microscope (Nikon, Seoul, Korea).

### 2.15 Immunohistochemistry (IHC)

Paraffin‐embedded tissue slides were deparaffinized with xylene, dipped in 100%, 95%, 80% and 70% ethanol for hydration and washed with tap water for 10 min. Heat‐induced epitope retrieval (HIER) was performed using Tris‐EDTA (10 mM Tris Base, 1 mM EDTA Solution, 0.05% Tween 20, pH 9.0). The tissue slides were stained with immunostained or H&E with antibodies at 4°C overnight. After washing with PBST (10% Tween 20 in PBS), the sections were treated with biotinylated goat anti‐mouse IgG or anti‐rabbit IgG antibody (1:200). After washing with PBS and treatment with ABC solution, a colour reaction was performed with 3,3′‐diaminobenzidine (Vector Laboratories, Burlingame, CA, USA). The nuclei were counterstained with haematoxylin for 3 min. After washing in tap water, the slides were dehydrated and mounted for observation using an IX71 microscope (Olympus, Seoul, Korea). The semi‐quantitative cytoplasmic and nuclear protein in stained slide calculated the optical density using an IHC Profiler.^35^ The infiltrated cells were counted. ZEB1‐positive cells infiltrated from the tumour margin in ZEB1‐stained mouse tissue and were counted at 100× using a microscope (BX50, Olympus, Seoul, Korea). The cohorts of patient tissue used for IHC are shown in Table S5.

### 2.16 Immunofluorescence staining

Paraffin‐embedded tissue slides were deparaffinized with xylene, dipped in 100%, 95%, 80% and 70% ethanol for hydration and washed with tap water for 10 min. HIER was performed using Tris‐EDTA. The tissue slides were immunostained with antibodies at 4°C overnight. After washing with PBST, the slides were treated with Alexa Fluor 546 conjugated secondary antibody, depending on the primary antibody at room temperature for 1 h. Cell nuclei were counterstained using DAPI and observed using an IX71 microscope. The cohorts of patient tissues used for immunofluorescence are shown in Table S5.

### 2.17 Gene set enrichment analysis (GSEA) and Kaplan–Meier analysis

GSEA was used to compare gene sets by diverse gene signature on Molecular Signature Database (MSigDB). MSLC‐positive and ‐negative patient microarray data analyzed in this study were previously published under the accession code GSE131837. Detailed information on patient cohorts is given in Table S6. For additional GSEA analysis, survival, subtype expression and two‐gene scatter plots were analyzed with the Prism software using The Cancer Genome Atlas (TCGA) lower grade glioma and glioblastoma (GBMLGG) dataset provided by UCSC Xena. REMBRANDT and TCGA survival were analyzed by dividing into high and low levels according to the median expression level of the indicated gene (www.betastasis.com).

### 2.18 Animal studies

The orthotopic tumour formed by GBM cells was co‐inoculated with MSLCs in male athymic nude mice (5–8‐week old) (Animal Inc., Central Lab, Seoul, Korea). The study was performed using mice maintained in adequate health for at least 1 week prior to the study in sterile microisolator cages. The mice were anaesthetized by intraperitoneal injection of a Zoletil (30 mg/kg; Virbac, Seoul, Korea) and xylazine (10 mg/kg; Bayer, Seoul, Korea) mixture. GBM cells (2 × 10^5^) alone or in combination with MSLCs were co‐injected at a 1:1 ratio to the right frontal lobe of the mouse brain using a Hamilton syringe at 0.5 μl/min (Dongwoo Science Co., Seoul, Korea) with a guide‐screw system (total four groups: X01 alone, X01+MSLC09‐03, X01+MSLC09‐03 shCD40 and X01+MSLC09‐03 neutralizing CD40L Ab; each group *n* = 6).^36^ At both 10 and 15 days, tail vein injection of CD40L neutralizing Ab (3 mg/kg, mabg‐h40l‐3, invivoGen, CA, USA) was carried out in the X01+MSLC09‐03 group. After 3 weeks, all four groups were sacrificed for IHC analysis.

### 2.19 Study ethics approval

Animal experimental procedures were carried out with the approval of the Institutional Animal Care and Use Committee (IACUC) of Severance Hospital of Yonsei University College of Medicine. The human studies were approved by the institutional review boards of Severance Hospital, Yonsei University College of Medicine (4‐2012‐0212, 4‐2014‐0649), and informed consent of the patient was obtained according to the Declaration of Helsinki.

### 2.20 Statistical analysis

All experimental values are reported as means and the error bars depict the standard deviation (SD). Values were compared using the unpaired Student's *t*‐test, and multivariate analysis was performed using analysis of variance (ANOVA). All statistical analyses were performed using the GraphPad Prism software (version 9.0). Statistical significance was set at *p*‐value <.05 (*p < .05, **p < .01, ***p < .001, ****p < .0001).

## 3 RESULTS

### 3.1 Tumour‐associated MSLCs modulated ECM and were involved in crosstalk with GBMs

**FIGURE 1:**
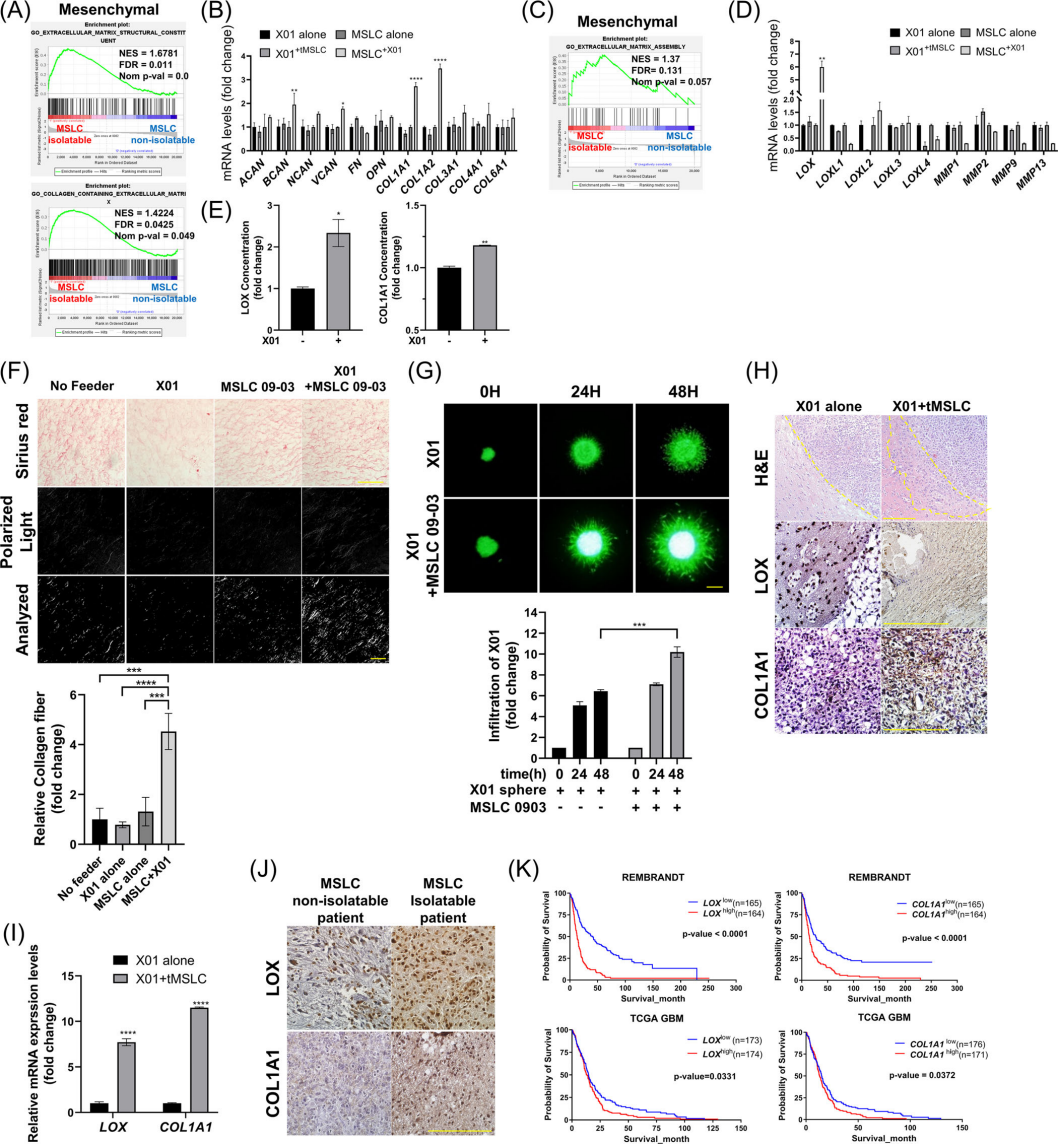
Glioblastoma (GBM) upregulated *LOX* and *COL1A1* in mesenchymal stem‐like cells (MSLCs), which promoted GBM cell infiltration. (A) Gene set enrichment analysis (GSEA) for extracellular matrix (ECM)‐related signature in MSLC‐isolatable and MSLC‐non‐isolatable patients. (B) Quantitative reverse transcription‐polymerase chain reaction (qRT‐PCR) of ECM molecules in MSLCs and X01 cells co‐cultured with each other. Normalization was performed from each X01 to X01+MSLC and MSLC to MSLC+X01. +X01 or +MSLC, designated by superscripts, indicates the cells that have been co‐cultured with MSLC and X01, respectively. (C) GSEA for ECM remodelling‐related signature for MSLC‐isolatable and MSLC‐non‐isolatable patients. (D) qRT‐PCR of ECM remodelling enzymes in MSLCs and X01 cells co‐cultured with each other. Normalization was performed from each X01 to X01+MSLC and MSLC to MSLC+X01. +X01 or +MSLC, designated by superscripts, indicates the cells that have been co‐cultured with MSLC and X01, respectively. (E) Enzyme‐linked immunosorbent assay of LOX and COL1A1 levels in MSLCs co‐cultured with X01 (*n* = 3). (F) Representative image of 3D collagen‐based matrix incubated with X01 and/or MSLCs, stained with Picrosirius red. Image: bright field on top, collagen fibre in the middle (polarized light) and analysis of polarized area at the bottom. Collagen fibre area = polarized area/total area (*n* = 4). Bright field scale bar: 100 μm; polarized light scale bar: 10 μm. (G) X01 spheroid infiltration within 3D collagen‐based matrix co‐cultured with MSLCs. Bottom graph shows calculation results of the infiltration area. Scale bar: 200 μm. (H) H&E staining and immunohistochemistry (IHC) of LOX and COL1A1 in mouse brain coronal section (*n* = 6 mice/group). Scale bar: 200 μm. (I) *LOX* and *COL1A1* expression levels in mouse brains injected with X01 alone and X01+MSLC (*n* = 6 mice/group). (J) IHC for LOX and COL1A1 in MSLC‐non‐isolatable and MSLC‐isolatable GBM patient specimen. Scale bar: 100 μm. (K) Kaplan–Meier survival curve of all patients with glioma (REMBRANDT) and GBM (The Cancer Genome Atlas; TCGA) with high or low *LOX* and *COL1A1* median expression. *p < .05, **p < .01, ***p < .001, ****p < .0001

Previously, we isolated human MSLCs from patients with glioma.^19^ These cells do not produce tumours and show MSC phenotype (morphology, cell surface marker and differentiation); furthermore, the survival of MSLC‐isolatable patients was lower than that of MSLC non‐isolatable patients.^20^ In addition, our previous study showed that MSLCs increased the infiltration of GBM cells by inducing mechanical shrinkage of ECM.^37^ Hence, we hypothesized that MSLC‐remodelled ECM (shrinkage, crosslinking, deposition, degradation) might increase the invasion of GBM cells.^37^, ^38^, ^39^ To confirm this hypothesis, we performed gene ontology (GO) analysis using the GSEA; the results did not show any substantial difference between the MSLC isolatable and MSLC non‐isolatable groups considering all patients with GBM; however, in the mesenchymal type, there was a high correlation in the collagen‐containing parts and ECM assembly, followed by the proneural type, and not in the classical type (Figure 1A,C; Figure S1A,B). These data were indicative of abundant ECM remodelling in the mesenchymal and proneural types in the presence of MSLCs. On the basis of these results, we analyzed the expressions of ECM molecules and ECM‐remodelled enzymes in GBM cells alone (X01; mesenchymal subtype^40^), MSLCs alone, and the co‐culture group. We observed increased *COL1A1*, *COL1A2* and *LOX* expressions in co‐cultured MSLCs (not GBM cells) compared to MSLCs alone (Figure 1B,D; Figure S1C,D). Bone marrow‐derived mesenchymal stem cells (BM‐MSCs) also increased *LOX* and *COL1A1* expressions when co‐cultured with X01 cells (Figure S1E). LOX and COL1A1 secretion levels in CM were significantly high when co‐cultured (Figure 1E). Moreover, *LOX* and *COL1A1* expressions increased in MSLCs when treated with CM from X01 (Figure S1F,G). Furthermore, other GBM CM could increase *LOX* and *COL1A1* expression in MSLCs (Figure S1H). In addition, LOX activity increased significantly when X01 and MSLCs were co‐cultured (Figure S1I).

Accordingly, we created a collagen‐based ECM mixture mimicking the in vivo ECM. ECM remodelling (collagen fibre formation), when cells were cultured in the ECM mixture, was analyzed using Picrosirius red staining and polarized light microscopy, and GBM cell invasion was analyzed using the 3D invasion assay. We observed that compared to GBM cells (X01 or GSC‐11; proneural subtype) or MSLCs alone, collagen fibre and invasion of GBM cells increased when GBM cells were co‐cultured with MSLCs (Figure 1F,G; Figure S1J,K).

Similar to the results of IHC, qRT‐PCR and H&E staining, *LOX* and *COL1A1* expressions and tumour infiltration increased in vivo when GBM cells (X01 cells) were co‐injected with MSLCs rather than when injected alone (Figure 1H,I). In the patient tissue, expressions of *LOX* and *COL1A1* were high in MSLC‐isolatable cases, as observed using IHC (Figure 1J). In addition, the data on the survival rate of patients with glioma from the REMBRANDT study and TCGA GBM databases showed that high expressions of *LOX* and *COL1A1* correlated with low survival rate (Figure 1K). These results suggest that GBM cells induce *LOX* and *COL1A1* expression and secretion by MSLCs, MSLC remodelled ECM, which leads to an increase in GBM infiltration potential. We hypothesized that there would be a paracrine loop between GBM cells and MSLCs.

### 3.2 LOX secreted from MSLCs increased GBM infiltration by remodelling the ECM

**FIGURE 2:**
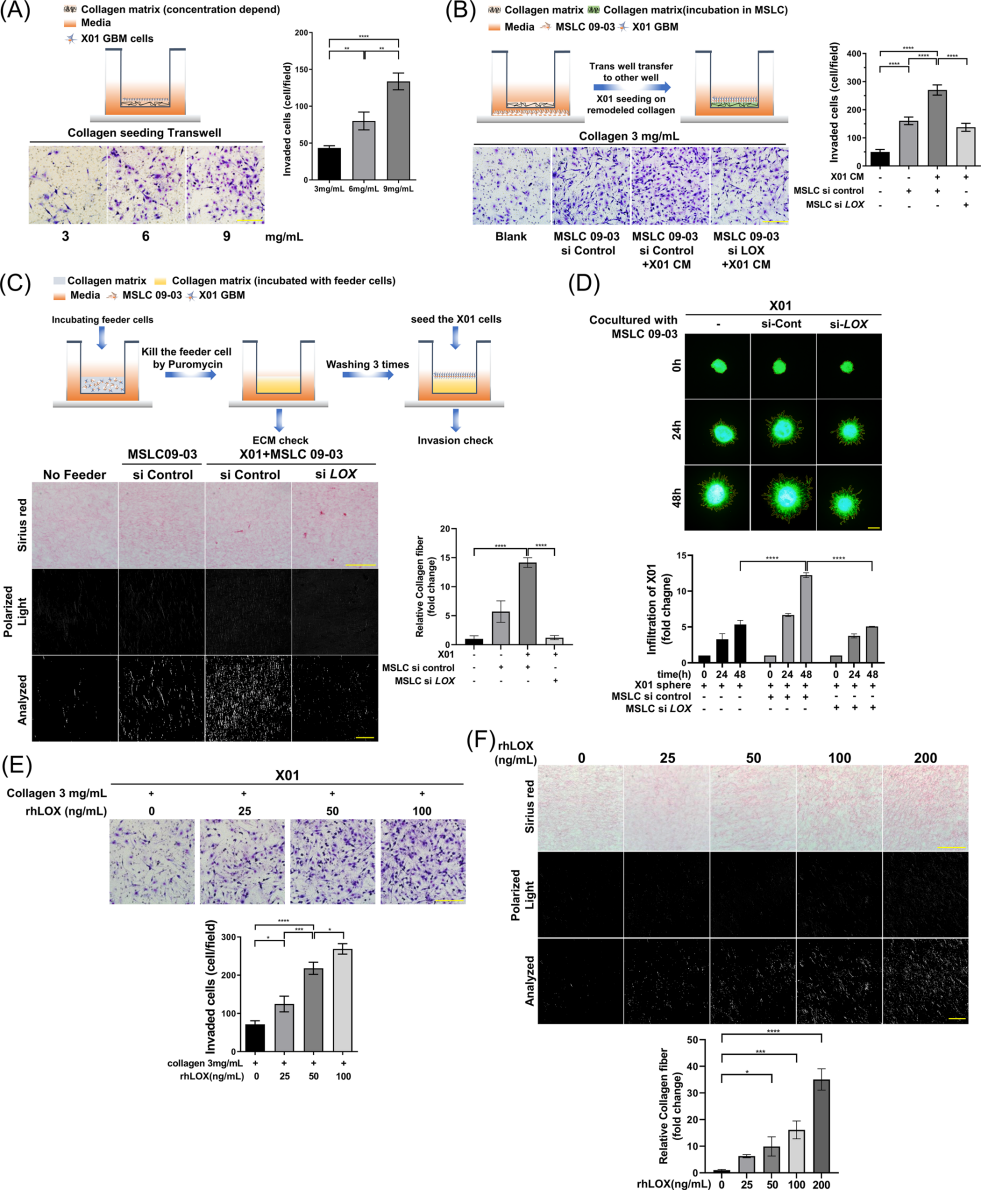
LOX promoted glioblastoma (GBM) infiltration by remodelling the extracellular matrix (ECM). (A) Schematic model (top) and collagen concentration‐dependent X01 GBM cell invasion assay. Scale bar: 200 μm. (B) Schematic model (top) and invasion of GBM cells after incubation in a transwell coated with 3 mg/ml collagen. Scale bar: 200 μm. (C) Schematic model (top) and representative image of 3D collagen‐based matrix pre‐incubated with X01 cells and/or MSLCs transfected with siRNA‐control or si‐*LOX*, stained with Picrosirius red. Image: bright field at the top, collagen fibre in the middle (polarized light), and analysis of polarized area at the bottom. Collagen fibre area = polarized area/total area (*n* = 4). Bright field scale bar: 100 μm; polarized light scale bar: 10 μm. (D) X01 spheroid infiltration in 3D collagen‐based matrix co‐cultured with MSLCs transfected with siRNA‐control or si‐*LOX*. Bottom graph shows calculation results of the infiltration area. Scale bar: 200 μm. (E) Invasion of GBM cells after pre‐incubation in DMEM treated with rhLOX in a concentration‐dependent manner in transwell coated with 3 mg/ml collagen. Scale bar: 200 μm. (F) Representative image of 3D collagen‐based matrix pre‐incubated with rhLOX in a concentration‐dependent manner, stained with Picrosirius red. Image: bright field (top), collagen fibre (mid, polarized light), and analysis of polarized area (bottom). Collagen fibre area = polarized area/total area (*n* = 4). Bright field scale bar: 100 μm; polarized light scale bar: 10 μm. *p < .05, **p < .01, ***p < .001, ****p < .0001

From previous results, we predicted that there was a paracrine loop between MSLCs and GBM cells, and we confirmed whether LOX and COL1A1 affected GBM invasion. In the transwell coated with collagen type 1, the invasiveness of X01 cells increased in a collagen concentration‐dependent manner (Figure 2A). Interestingly, transwells incubated with MSLCs showed an increase in GBM cell invasion; X01 CM‐treated MSLCs showed more increased invasion than MSLCs alone, which was, however, not observed when *LOX* was knocked down (Figure 2B; Figure S2A–B). Similarly, the collagen matrix was more remodelled in the presence of MSLCs or under co‐incubation conditions than in the absence of MSLCs. However, ECM remodelling and GBM infiltration in the ECM decreased when *LOX* was knocked down (Figure 2C,D; Figure S2C,D).

Next, we investigated whether treatment with rhLOX increased GBM cell invasion and infiltration into the ECM. First, we performed a LOX activity assay on rhLOX to determine the activity of LOX. The activity of rhLOX increased in a concentration‐dependent manner, and the decrease in activity by BAPN was confirmed (Figure S2E,F). As anticipated, invasion into collagen‐coated transwell, collagen fibre formation and GBM infiltration on ECM increased by rhLOX in a concentration‐dependent manner in the presence of 3 and 6 mg/ml collagen (Figure 2E,F; Figure S2G–I). These results suggested that GBM invasion was affected by LOX and COL1A1 in a concentration‐dependent manner.

### 3.3 GBMs contributed to regulation of LOX expression of MSLCs via CD40L

**FIGURE 3:**
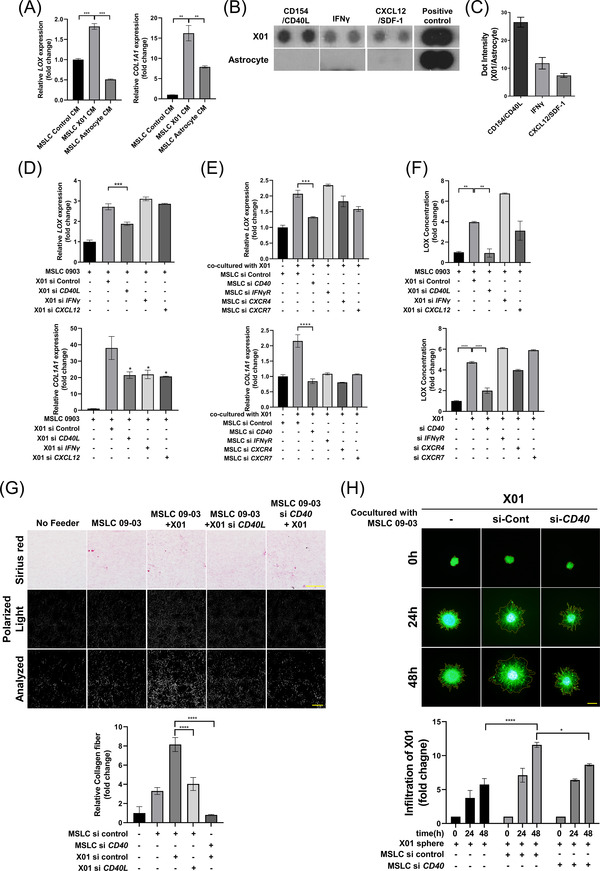
Glioblastoma (GBM) secretes CD40L to upregulate LOX expression via CD40 in MSLCs. (A) Quantitative reverse transcription‐polymerase chain reaction (qRT‐PCR) analysis in MSLC treatment control conditioned media (CM), X01 CM or astrocyte CM. (B) Cytokine array of X01 CM or astrocyte CM as indicated (*n* = 2). (C) Densitometry analysis of cytokine array shown in (B). (D) qRT‐PCR for *LOX* and *COL1A1* in MSLCs co‐cultured with X01 cells depleted of cytokine genes using siRNA. (E) qRT‐PCR for *LOX* and *COL1A1* in MSLCs depleted of cytokine receptor gene using siRNA co‐cultured with X01. (F) Enzyme‐linked immunosorbent assay of LOX level in CM from (D) and (E). (G) Representative image of 3D collagen‐based matrix pre‐incubated with X01 cells transfected with siRNA‐control or si‐*CD40L* and/or MSLCs transfected with siRNA‐control or si‐*CD40* stained with Picrosirius red. Image: bright field (top), collagen fibre (mid, polarized light), and analysis of polarized area (bottom). Collagen fibre area = polarized area/total area (*n* = 4). Bright field scale bar: 100 μm; polarized light scale bar: 10 μm. (H) X01 spheroid infiltration in 3D collagen‐based matrix co‐cultured with MSLCs transfected with siRNA‐control or si‐*CD40*. Bottom graph shows calculation results of the infiltration area. Scale bar: 200 μm. *p < .05, **p < .01, ***p < .001, ****p < .0001

The above data showed that GBM invasiveness was directly affected by LOX and COL1A1 in a concentration‐dependent manner, and LOX and COL1A1 expression increased remarkably in MSLCs when co‐cultured with GBM cells. Therefore, we predicted that MSLCs might be affected by soluble factors in a paracrine manner, and analyzed whether the expressions of LOX and COL1A1 were actually influenced by any soluble factor secreted by GBMs by treating MSLCs with the CM of GBM and normal astrocytes. Astrocytes are the most common glial cells in the central nervous system.^41^, ^42^ Indeed, as shown by qRT‐PCR and Western blotting, *LOX* expression in MSLCs increased when treated with the CM of GBM cells, but not that of astrocytes (Figure 3A; Figure S3A). However, *COL1A1* expression significantly increased in both astrocytes and X01 CM. Next, using cytokine array analysis, we detected a higher expression of CD40L, IFN‐γ and CXCL12 in X01 GBM cells than in astrocytes (Figure 3B,C; Figure S3B). The expression level of *CD40L* was not affected by serum, and the secretion and expression of *CD40L* in several GBM cells could be confirmed (Figure S3C–F). Furthermore, on the basis of previous data, each secretory factor was knocked down in GBM cells, which was then co‐cultured with MSLCs. The downregulation of *CD40L* and its receptor *CD40* effectively abolished the enhancement of *LOX* expression in MSLCs; however, downregulation of other secretory factors (*IFN‐γ* and *CXCL12*) and their receptors (*IFN‐γR*, *CXCR4* and *CXCR7*) did not effectively abolish increased *LOX* expression (Figure 3D–F; Figure S3G–I). Similarly, upregulation of LOX by X01 CM treatment in MSLCs was inhibited only when *CD40L* and *CD40* were knocked down in GBM cells and MSLCs (Figure S3J–L). However, COL1A1 was regulated by CD40L, IFN‐γ, CXCL12, CD40, IFN‐γR, CXCR4 and CXCR7. Collagen fibre formation and infiltration decreased when *CD40* was knocked down in MSLCs and *CD40L* in X01 cells (Figure 3G; Figure S3M). The 3D spheroid invasion assay also revealed a decrease in X01 and GSC‐11 cell invasion when *CD40* was knocked down in MSLCs (Figure 3H; Figure S3N). These results suggested that GBM cells secreted CD40L to induce LOX in MSLCs via CD40, thereby increasing the infiltration of GBM cells through ECM remodelling.

### 3.4 CD40L increased LOX expression via CD40‐mediated nuclear translocation of NF‐κB2 in MSLCs

**FIGURE 4:**
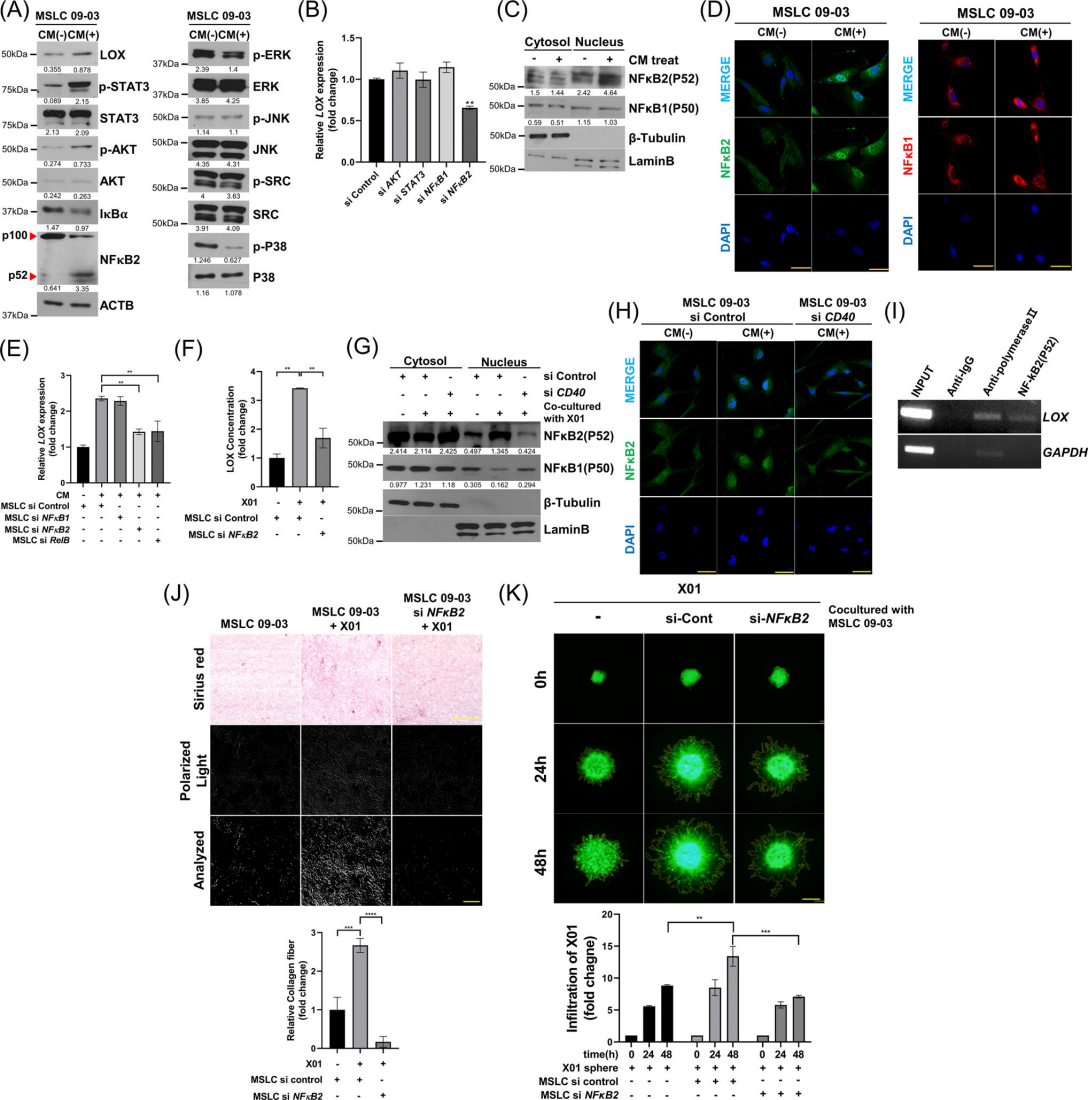
CD40L increased LOX expression via CD40‐mediated NF‐κB2 nucleus translocation. (A) Western blot analysis to determine CD40 downstream effector activation status in MSLCs treated with control conditioned medium (CM) or X01 CM. (B) Quantitative reverse transcription‐polymerase chain reaction (qRT‐PCR) of *LOX* expression in MSLCs transfected with siRNA as indicated. (C) Western blot analysis of the nucleus translocation of NF‐κB1 or NF‐κB2 by X01 CM in the cytosol‐nuclear fraction of MSLCs. (D) Immunocytochemistry in MSLCs and nucleus translocation of NF‐κB1 or NF‐κB2 by X01 CM. Scale bar: 50 μm. (E) qRT‐PCR of *LOX* expression in MSLCs treated with X01 CM and transfected with siRNA as indicated. (F) Enzyme‐linked immunosorbent assay of LOX in CM after co‐culture with each transfected cell type. (G) Western blots of fractionated lysates of MSLCs transfected with siRNA‐control or si‐*CD40* after co‐culture with X01. (H) Nuclear translocation of NF‐κB2 in MSLC siRNA‐control or MSLC si‐*CD40* treated or not treated with X01 CM. Scale bar: 50 μm. (I) Chromatin immunoprecipitation for assessing NF‐κB2 binding to *LOX* promoter in MSLCs. (J) Representative image of 3D collagen‐based matrix pre‐incubated with MSLCs transfected with siRNA‐control or si‐*NF‐κB2* and/or X01 cells stained with Picrosirius red. Image: bright field (top), collagen fibre (mid, polarized light), and analysis of polarized area (bottom). Bottom graph shows collagen fibre area = polarized area/total area (*n* = 4). Bright field scale bar: 100 μm; polarized light scale bar: 10 μm. (K) X01 spheroid infiltration in 3D collagen‐based matrix co‐cultured with MSLCs transfected with siRNA‐control or si‐*NF‐κB2*. Bottom graph shows calculation results of the infiltration area. Scale bar: 200 μm. **p < .01, ***p < .001, ****p < .0001

In Section 3.3, it was confirmed that LOX expression of MSLC was upregulated through CD40L expressed in GBM cells. We used rhCD40L protein to determine the expression of LOX in MSLCs; the results showed that rhCD40L increased LOX expression in MSLCs in a concentration‐dependent manner (Figure S4A,B). CD40 predominantly signals via NF‐κB, MAPK, STAT, PI3K, and SRC have also been reported to be involved.^43^, ^44^ Hence, we assessed the activation status of several signalling pathways after treatment of MSLCs with X01 CM or rhCD40L. The results showed that AKT, STAT3, NF‐κB1 and NF‐κB2 were the main activated signalling pathways, and that only NF‐κB2 regulated LOX expression (Figure 4A,B; Figure S4C,G). To confirm the nuclear translocation of NF‐κB1 and NF‐κB2 in MSLCs after CM and rhCD40L treatment, we performed nuclear‐cytoplasmic fractionation and immunocytochemistry. It was confirmed that the nuclear translocation of NF‐κB2 (not NF‐κB1) was increased by CM and rhCD40L (Figure 4C,D; Figure S4D,E). To further confirm the specificity of CM and rhCD40L, we knocked down *CD40*, *NF*‐*κB1*, *NF*‐*κB2* and *RelB* in MSLCs. When *NF*‐*κB2* and the co‐transcription factor *RelB* were depleted, *LOX* expression did not increase even after treatment with X01 CM (Figure 4E,F; Figure S4F,G); the nuclear translocation of NFκ‐B2 decreased when *CD40* was knocked down (Figure 4G,H; Figure S4H). Furthermore, NF‐κB2 was found to bind to the promoter of *LOX* in a ChIP assay in MSLCs (Figure 4I). Finally, we confirmed that NF‐κB2 inhibition substantially reduced the collagen fibre formation of ECM and that the increase in collagen fibre and infiltration observed upon co‐culture of X01 cells and MSLCs was abolished when NF‐κB2 was depleted in MSLCs (Figure 4J,K; Figure S4I,J).

Previously, it has been reported that LOX remodels the ECM, and also regulates intranuclear transcription.^45^, ^46^ Therefore, we investigated whether LOX directly regulates CD40L and CD40 expressions or not, and we confirmed that LOX did not regulate CD40L and CD40 expressions (Figure S5A). However, NF‐κB2 appeared to be related to the regulation of CD40 and CD40L expressions; the results of the ChIP assay also confirmed the binding of NF‐κB2 to promoter regions of *CD40* and *CD40L*. This suggests that the cue of feedback loop in MSLC was stimulated by CD40L secreted by GBM (Figure S5B–D). Also in BM‐MSCs, CD40L and CD40 were more expressed when co‐cultured with X01 than without (Figure S5E). Thus, CD40L that activates the CD40/NF‐κB2 signalling axis in MSLCs promotes ECM remodelling by increasing LOX expression and activating a feedback loop.

### 3.5 CD40L‐neutralizing Ab inhibited GBM infiltration by inhibiting CD40 signalling of MSLC in vivo

**FIGURE 5:**
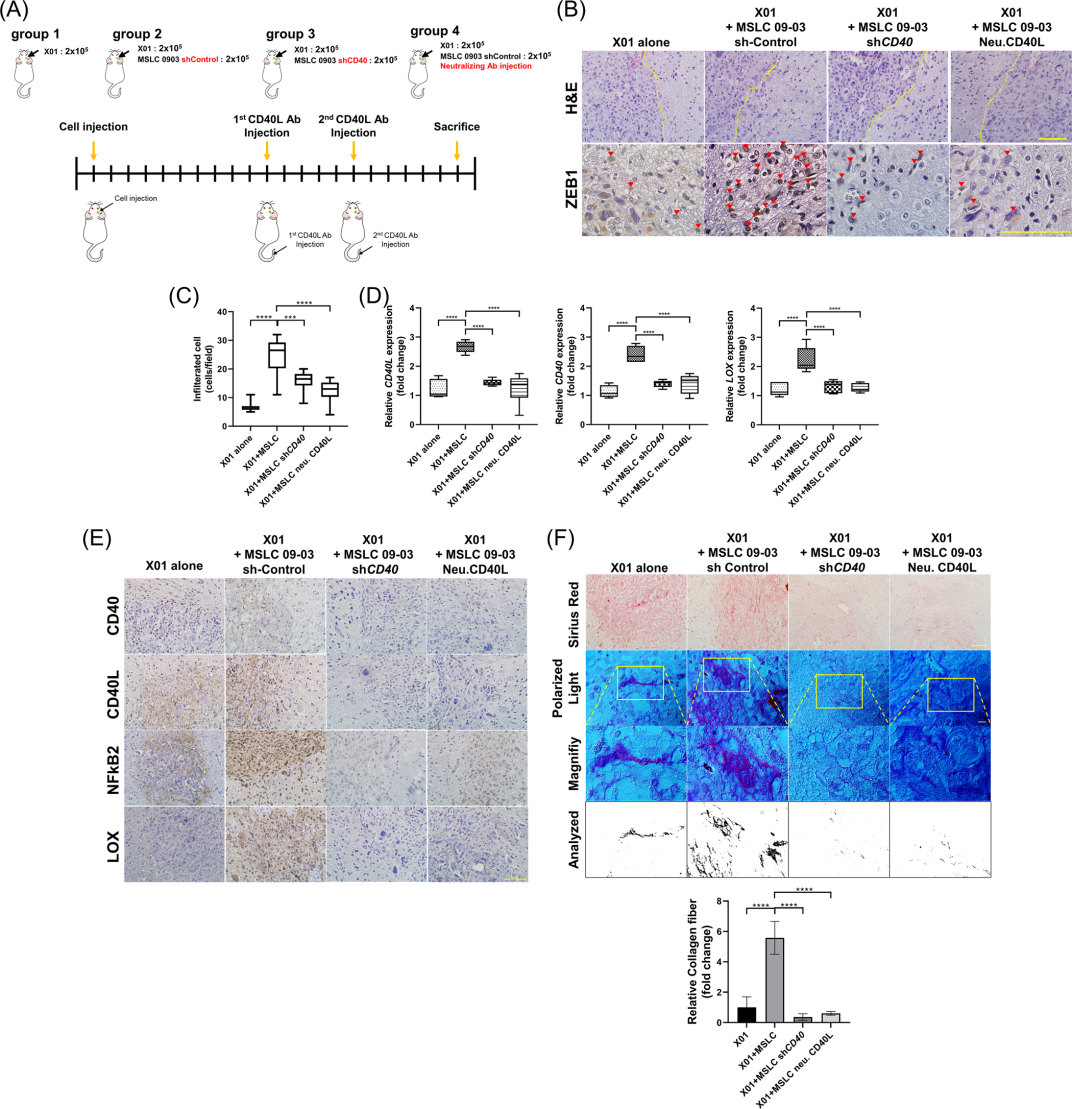
CD40L neutralizing antibody and knockdown of CD40 suppressed ECM remodelling and GBM infiltration. (A) Schematic illustration of GBM model generation and the overall therapeutic procedure for orthotopic xenograft animal model. (B) H&E and ZEB1 staining of coronally sectioned mouse brain. Yellow dash represents tumour margin. Red triangles represent ZEB1‐positive infiltration cells (*n* = 6 mouse/group). Scale bar: 100 μm. (C) The number of ZEB1‐positive cells infiltrated outside the tumour margin in (B). (D) Quantitative reverse transcription‐polymerase chain reaction (qRT‐PCR) of *CD40L*, *CD40* and *LOX* expression in mouse brain tissue (*n* = 3 mouse/group). (E) IHC of CD40, CD40L, NFκB2 and LOX in the indicated groups (*n* = 6 mouse/group). Scale bar: 100 μm. (F) Representative image of Picrosirius red‐stained mouse brain tissue for each xenograft group. Collagen fibre area = polarized area/total area (*n* = 6 mouse/group). Collagen fibre area (right) = polarized area/total area (*n* = 6 mouse/group). Bright field scale bar: 100 μm; polarized light scale bar: 10 μm. ***p < .001, ****p < .0001

We have demonstrated that X01‐secreted CD40L promotes ECM remodelling in MSLCs, and we presumed that CD40L‐neutralizing antibodies could inhibit ECM remodelling. We observed that *CD40L*, *CD40* and *LOX* expressions were suppressed in a concentration‐dependent manner in vitro with a CD40L‐neutralizing antibody (Figure S6A). For further in vivo analysis, a tumour was generated by transducing sh*CD40* or shcontrol into MSLCs and orthotopically co‐inoculating the knocked down cells along with X01 cells into mouse brain. Furthermore, to block CD40L, a neutralizing CD40L antibody was injected via the tail vein of mouse (Figure 5A; Figure S6B). Upon tumour formation, GBM cells co‐inoculated with MSLC‐invaded areas adjacent to the brain more than GBM cells alone. However, *CD40* depletion in MSLCs and blocking CD40L through neutralizing antibody decreased GBM cell infiltration (Figure 5B,C). In addition, *CD40L*, *CD40* and *LOX* expressions were more increased in the X01+MSLC sh control group than in the X01 alone group. However, X01+MSLC sh CD40 and X01+MSLC neutralizing CD40L group showed decreased CD40L, CD40 and LOX expression than X01+MSLC sh control group (Figure 5D,E; Figure S6C). IHC analysis also showed reduction in target gene expression and NF‐κB2 nuclear translocation in *CD40*‐depleted MSLCs and after CD40L neutralizing antibody treatment. The Picrosirius red‐stained mouse brain tissue sections were examined under non‐polarized and polarized light. As expected, the tissues co‐inoculated with MSLCs and GBM cells showed high collagen fibre formation, whereas the *CD40*‐deficient MSLCs and those treated with the neutralizing CD40L antibody showed substantially reduced collagen fibre formation (Figure 5F). We performed a co‐immunofluorescence assay by combining GBM (CD105^–^, CD44^+^ and CD45^+^) and MSLC (CD105^+^, CD44^+^ and CD45^–^) to confirm the expressions of CD40L and CD40 in GBM cells and MSLCs in the in vivo mouse tissue sample. X01 alone tissue showed CD44^+^CD45^+^, CD45^+^CD40L^+^ and CD45^+^CD40^+^ cells, but not CD105^+^ cells. In the X01+MSLC co‐inoculation tissue, CD44^+^CD45^–^, CD45^–^CD40L^+^, CD45^–^CD40^+^ and CD105^+^CD44^+^ cells were detected. These data reveal the expressions of CD40L and CD40 in GBM and MSLC cells (Figure S6D). It was possible to confirm the co‐presence of MSLC in MSLC‐isolatable GBM patients via IHC (Figure S6E). Furthermore, MSLC non‐isolatable patients showed low CD40 expression and MSLC isolatable patients showed high CD40 expression (Figure S6F). These data are correlated in Figure S5A–E. The results suggested that when MSLCs are co‐present with GBM, CD40L blockade possibly inhibits GBM infiltration.

### 3.6 MSLCs residing in GBM tumours correlated with clinical outcome of patients with GBMs

**FIGURE 6:**
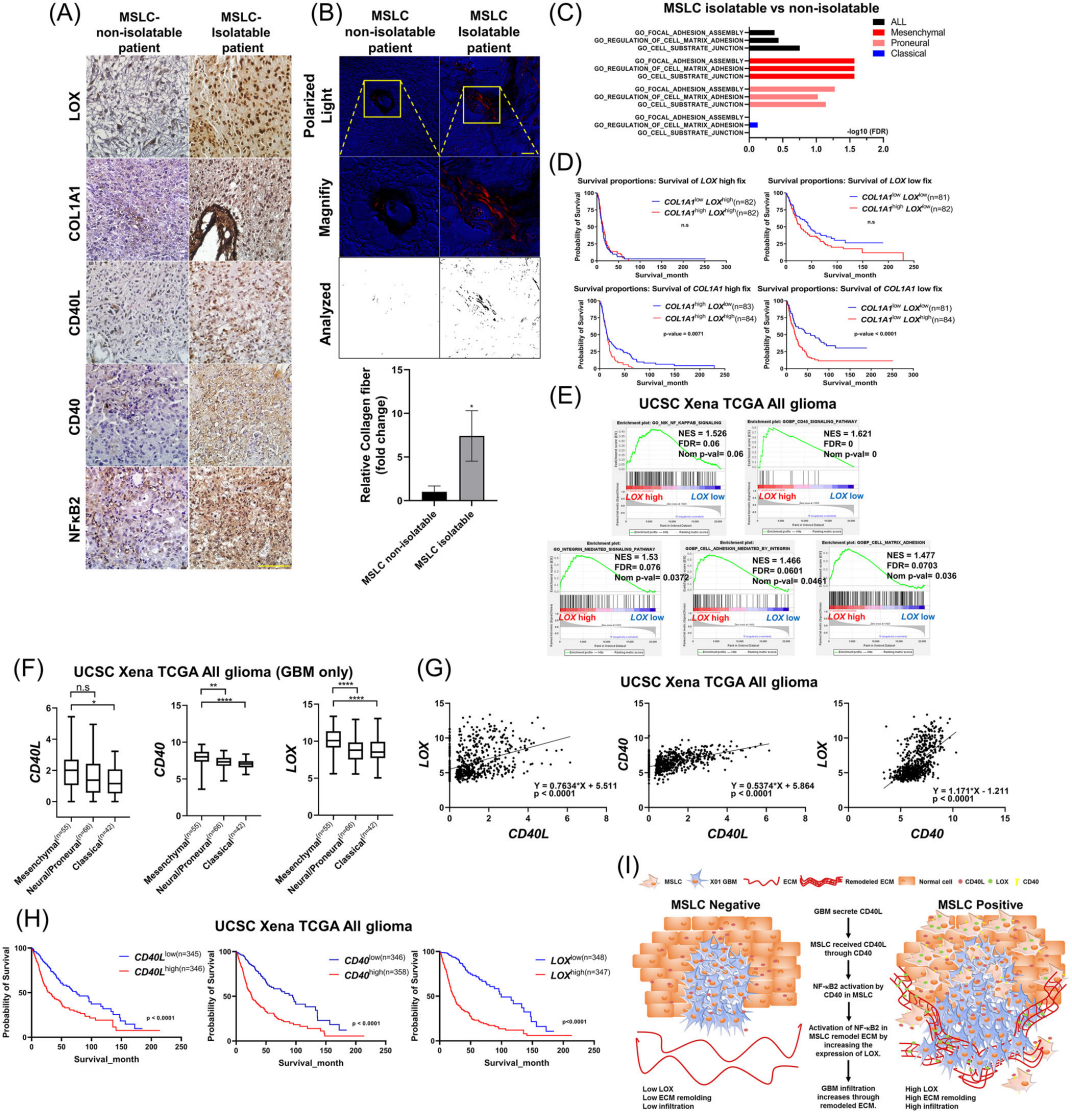
Correlation between tumour MSLC‐isolatable patients and clinical outcome. (A) IHC of LOX, COL1A1, CD40L, CD40 and NF‐κB2 in MSLC‐isolatable (*n* = 4) and MSLC‐non‐isolatable (*n* = 4) patient samples. Scale bar: 100 μm. (B) Picrosirius red‐stained MSLC‐isolatable (*n* = 3) and MSLC‐non‐isolatable (*n* = 3) GBM patient samples. Image: collagen fibre (top, polarized light) and analysis of polarized area (bottom). Graph shows collagen fibre area = polarized area/total area (*n* = 3). (C) Gene set enrichment analysis (GSEA) of MSLC‐isolatable and non‐isolatable patients. Analysis of gene set related to ECM‐cell adhesion by each GBM subtype. (D) Kaplan–Meier survival curves of all glioma patients (REMBRANDT) with fixed high or low *LOX* and *COL1A1* median expression. (E) GSEA of all GBMLGG patients with high and low median *LOX* expression. (F) GBM subtype‐specific expression of the indicated genes in patients with GBM in The Cancer Genome Atlas (TCGA). (G) Two‐gene scatter plots of the indicated genes in patients with glioma in TCGA. (H) Kaplan–Meier survival curves of all glioma patients (TCGA) with high or low indicated median expression of gene. (I) Schematic summarizing promotion of ECM remodelling and GBM infiltration by GBM‐educated MSLCs in the tumour microenvironment. n.s. = not significant, *p < .05, **p < .01, ****p < .0001

CD40L, CD40, COL1A1 and LOX expressions, activation of NF‐κB2 and collagen fibre formation were found to be higher in MSLC‐isolatable patients than in MSLC‐non‐isolatable patients, as shown previously (Figure 6A,B; Figure S7A). GSEA was performed to confirm the association of this enhanced expression with ECM in MSLC‐isolatable patients. Results showed that the presence of MSLCs in the mesenchymal and proneural types correlated positively with the gene set related to crosstalk with the ECM (Figure 6C; Figure S7B). Further analysis was performed using the REMBRANDT database. *LOX* and *COL1A1* expression levels were fixed as *LOX* ^high^, *LOX* ^low^, *COL1A1* ^high^ and *COL1A1* ^low^ (four groups), and each group was divided again according to the *LOX* or *COL1A1* expression as low and high. In *LOX* ^high^ or *LOX* ^low^, fixed group (top) was not significant by *COL1A1* expression; however, in *COL1A1* ^high^ or *COL1A1* ^low^, fixed group (bottom) was significant by *LOX* expression. These results reveal that *LOX* is associated with lower patient survival than *COL1A1* (Figure 6D). Next, GSEA was performed to examine the crosstalk between the activation of NF‐κB2 signalling pathway and ECM using the TCGA GBMLGG datasets available in the UCSC Xena browser. The data were divided based on LOX expression. We found that the higher the expression of LOX, the higher the CD40 signalling pathway, activation of NF‐κB2 and crosstalk with the ECM in the same GBMLGG dataset (Figure 6E). In addition, the expressions of *CD40L*, *CD40* and *LOX* increased with the tumour grade in all patients (Figure S7C), and higher expressions of the target genes were observed in the mesenchymal type (Figure 6F). As shown in previous sections, increase in LOX expression correlated positively with the expressions of CD40L and CD40, and the same result was confirmed using a two‐gene scatter plot in the GBMLGG set (Figure 6G). In the same GBMLGG dataset, high expressions of target genes associated remarkably with shorter survival rates. However, survival rates were not significant in grade 4 patients (Figure 6H, Figure S7D). Because, high expression of *CD40L*, *CD40* and *LOX* may reflect tumour malignancy in GBMLGG dataset, thus CD40L, CD40 and LOX expressions are associated with patient clinical outcomes.

## 4 DISCUSSION

The TME has been emerging as a hotspot for novel targets that can be used for developing therapies for several types of cancer. MSCs are present within the tumour in patients with GBM.^19^, ^20^ Accumulating evidence suggests that MSCs mainly play a tumour‐supportive role, and the cellular and molecular mechanisms via which MSCs regulate cancer invasion have been studied. However, whether the immunosuppressive properties of MSCs can be used for therapeutic and clinical applications remains controversial. Tumours may reprogramme MSCs to recruit them to the tumour site, where they play a tumour‐supportive role via secretion of exosomes, IL‐6, SDF‐1, PDGF and HGF in several cancers.^47^, ^48^, ^49^, ^50^ In our previous study, MSLC‐isolatable patients showed lower survival rates than that of MSLC‐non‐isolatable patients. In this study, we demonstrated that the MSLCs present in GBM are targeted by CD40L, which is secreted by the GBM cells. A previous study has reported that the promotion of CD40L/CD40 expression in GBM can induce immune stimulation and anti‐tumour responses.^51^ CD40L/CD40 has been attracting attention as a next‐generation immune checkpoint protein. However, the use of a single ICB for the treatment of GBM has been unsuccessful, and recent studies are underway to overcome this through ICB–chemical drug combination treatments.^7^, ^52^ In this context, ICB still focuses only on immune cells and excludes the effects on several other normal cells present in TME. As such, our results suggest the possibility that not only the activation of immune cells but also the effects on other normal cells constituting the GBM TME should be considered.

During cancer progression, the ECM is consistently remodelled, resulting in ECM stiffness in the TME.^10^, ^11^ Increase in ECM stiffness plays a critical role in tumour growth, invasion and metastasis via biochemical and biomechanical mechanisms. In accordance with this notion, we demonstrated that GBM cells co‐cultured with MSLCs became more invasive in the 3D culture system and spheroid assay. In addition, compared to MSLCs alone, GBM‐educated MSLCs secreted more LOX and remodelled the collagen fibre of the ECM. Analysis of the survival of patients with glioma expressing LOX or COL1A1 indicated that high expressions of these genes correlated with poor outcomes. Importantly, high expression of LOX was directly involved in the survival of patients irrespective of the expression level of COL1A1, suggesting that ECM stiffness is more important than accumulation of ECM molecules for patient survival. While we mainly confirmed the increased expressions of LOX and COL1A1 by MSLCs recruited to GBM TME, Chen et al. have demonstrated that GBM‐derived LOX increased macrophage recruitment, which promoted angiogenesis and survival of GBM itself.^53^ These results reveal that LOX secreted from GBM‐educated MSLCs or GBMs can act as cues for a tumour‐promoting microenvironment.

GBM cells are characterized by high expressions of inflammatory factors and tumorigenic genes and generally crosstalk with neighbouring cells in a paracrine manner. In our study, we showed that the MSLCs present in the GBM microenvironment induced collagen fibre formation of ECM via the CD40L/CD40 signalling pathway and promoted the invasion of GBM cells. Interestingly, we observed that patient‐derived X01 cells showed higher CD40L expression and secretion than that of astrocytes. Secreted CD40L bind to CD40 on the surface of MSLCs and reprogramme them to secrete LOX via NF‐κB2 signalling, which acted as a downstream mediator. However, when the expression of LOX was suppressed in CD40‐disrupted MSLCs, infiltration of GBM cells was inhibited in an in vitro 3D culture system. Furthermore, compared to that in mice inoculated with GBM cells alone, orthotopic co‐inoculation of GBM cells and MSLCs promoted GBM cell infiltration and ECM remodelling in mice. In contrast, co‐inoculation of GBM cells and CD40‐disrupted MSLCs and injection of a CD40L neutralizing antibody inhibited GBM cell infiltration and collagen fibre formation.

We focused on the inhibition of GBM infiltration that was promoted when MSLCs were present in the TME. According to our study results, CD40L neutralizing immunotherapy can yield positive results in patient survival when MSLCs are present in the TME. However, considering the in vivo function of CD40L, it will be necessary to conduct additional studies and mouse survival experiments with immune cells and MSLC in TME before the application of our study to clinical trials. We confirmed that high expressions of CD40L/CD40/LOX reflect a low patient survival rate through the TCGA GBMLGG and REMBRANDT databases. However, regarding bulk‐seq currently used for analysis, it seems that the need for single‐cell seq is necessary due to the need to check the presence or absence of MSLC in glioma and GBM patients and to analyze the expressions of GBM cells and MSLCs, respectively. Nevertheless, inhibition of CD40L can inhibit the infiltration of GBM cells into normal brain tissues in the GBM environment in which MSLCs exist, and can be expected to facilitate surgical resection and positively affect the survival rate of patients.

## 5 CONCLUSION

This study identified the mechanistic insights of MSLCs for the ECM remodelling of the GBM pro‐infiltrative TME. Intriguingly, CD40L secreted from GBM binds to CD40 on the MSLC surface and reprogrammes using the NF‐κB2 signalling axis to secrete LOX (Figure 6I). Importantly, blocking CD40L suppressed ECM remodelling and GBM infiltration. Our findings give us an insight into a potential therapeutic target that can be used for suppressing GBM infiltration and the easy surgical resection to suppress recurrence.

## CONFLICT OF INTEREST

The authors declare that there is no potential conflict of interest.

## Supporting information



ctm2997‐sup‐0001‐SuppMat.docx

ctm2997‐sup‐0002‐FigureS1.tif

ctm2997‐sup‐0003‐FigureS2.tif

ctm2997‐sup‐0004‐FigureS3.tif

ctm2997‐sup‐0005‐FigureS4.tif

ctm2997‐sup‐0006‐FigureS5.tif

ctm2997‐sup‐0007‐FigureS6.tif

ctm2997‐sup‐0008‐FigureS7.tif
