# Supplementary material for: Glioblastoma‐educated mesenchymal stem‐like cells promote glioblastoma infiltration via extracellular matrix remodelling in the tumour microenvironment
Source: Clin Transl Med. 2022 Jul 31;12(8):e997. doi: 10.1002/ctm2.997 (PMC9339241; doi:10.1002/ctm2.997)
Supplement: Supplementary file 1 — ctm2997‐sup‐0001‐SuppMat.docx [file CTM2-12-e997-s008.docx]

Supporting Information

**GBM-educated Mesenchymal stem-like cells promote GBM infiltration through ECM remodeling in tumor microenvironment**

Seung-Mo Kim^1,†^, Eun-Jung Lim^1,4^, Ki-Chun Yoo^,1,3^, Yi Zhao^1^, Jae-Hyeok Kang^1^, Eun-Ji Lim^1^, Incheol Shin^1^, Seok-Gu Kang^2^, Han Woong Lim^5^, Su-Jae Lee^1,6^*

^1^Department of Life Science, Research Institute for Natural Sciences, Hanyang University, Seoul, Korea.

^2^Department of Neurosurgery, Brain Tumor Center, Severance Hospital, Yonsei University College of Medicine, Seoul 03722, Korea

^3^Department of Lymphoma and Myeloma, Division of Cancer Medicine, Center for Cancer Immunology Research, The University of Texas MD Anderson Cancer Center, Houston, TX 77030, USA.

^4^Memorial Sloan Kettering, Cancer Center, New York, NY 10065, USA

^5^Department of Ophthalmology, Hanyang University Hospital, Hanyang University College of Medicine, Seoul, Korea

^6^Fibrosis and Cancer Targeting Biotechnology, FNCT Biotech, Seoul 04626, Korea

^†^Seung-Mo Kim was the main contributor to this work.

***Correspondence should be addressed to:** Su-Jae Lee, Ph.D

Laboratory of Molecular Biochemistry, Department of Life Science, Hanyang University, 222 Wangsimni-ro, Seongdong-gu, Seoul 04763, Korea.

Phone: 82-2-2220-2557; Fax: 82-2-2299-0762;

E-mail: sj0420@hanyang.ac.kr

**This Word file includes:**

Figures. S1 to S7

Tables S1 to S6


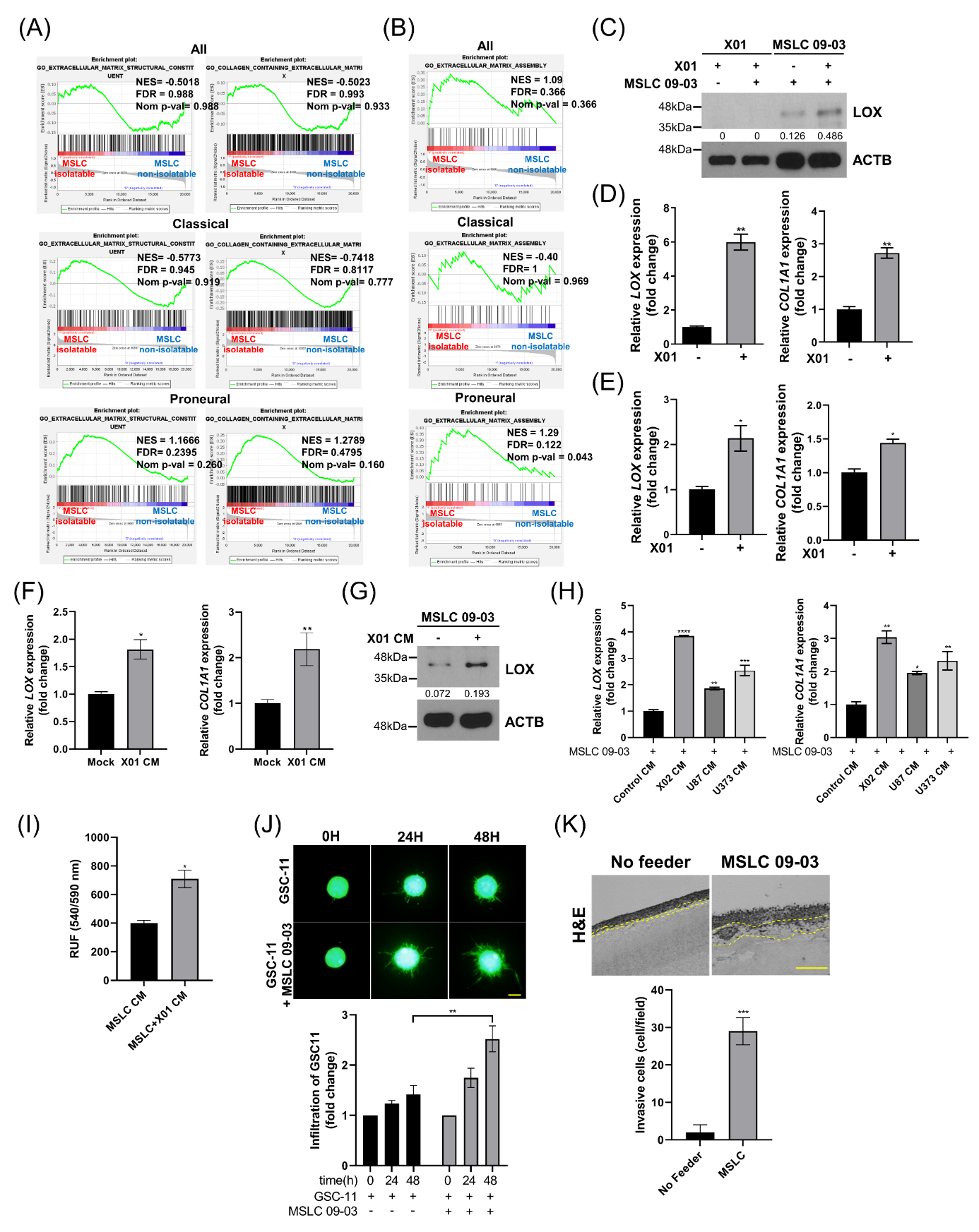


**Figure S1:** The expression of LOX and COL1A1 and GBM infiltration were increased, when GBM and MSLC were co-cultured.

(A) GSEA analysis for ECM molecule related signature in MSLC isolatable and MSLC non-isolatable patients for all patients, proneural, and classical subtype.

(B) GSEA analysis for ECM remodeling related signature in MSLC isolatable and MSLC non-isolatable patients for all patients, proneural, and classical subtype.

(C) Western blot of X01 and MSLC in alone and co-culture.

(D) Highlight of *LOX* and *COL1A1* mRNA expression for main figure (**B**) and (**D**).

(E) qRT-PCR of *LOX* and *COL1A1*expression in BM-MSCs after co-cultured with X01.

(F) qRT-PCR of *LOX* and *COL1A1* expression in MSLCs after treat X01 CM.

(G) Western blot of LOX expression in MSLC after treat X01 CM.

(H) qRT-PCR of *LOX* and *COL1A1* expression in MSLCs after treating indicated GBM cells CM.

(I) LOX activity assay in CM after co-cultured with each cells.

(J) GSC-11 spheroid cells infiltration with 3D collagen-based matrix co-cultured with MSLCs. Right graph shows calculation results of the infiltration area. Scale bar 200 µm.

(K) Hematoxylin and eosin (H&E) staining image and number of X01 GBM cells that infiltrated into collagen-based matrix pre-incubated with MSLC 09-03. Scale bar, 200 µm.

**P* < 0.05, ***P* < 0.01, ****P* < 0.001, *****P* < 0.0001.


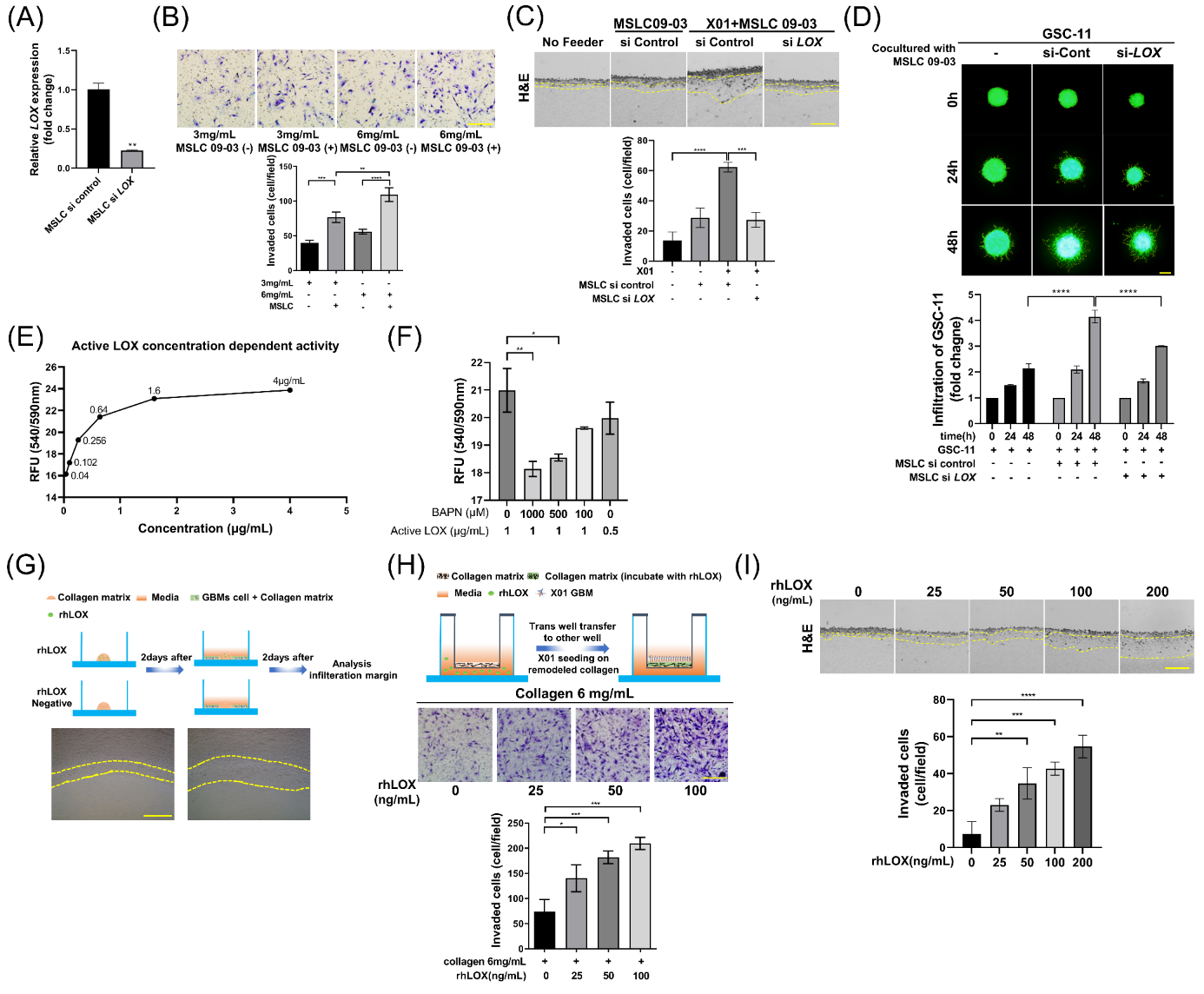


**Figure S2:** COL1A1 and LOX promote GBM infiltration.

(A) Transfection of siRNA knockdown efficiency of *LOX* expression by qRT-PCR in MSLCs.

(B) Invasion of GBM cells after incubation with MSLCs in a transwell coated with 3, 6mg/mL collagen. Scale bar 200 µm.

(C) H&E staining image and number of X01 GBM cells that infiltrated into the collagen-based matrix pre-incubated with X01 and/or MSLCs transfected with siRNA-control or si-*LOX*. Scale bar, 200 µm.

(D) GSC-11 spheroid cells infiltration with 3D collagen-based matrix co-cultured with MSLCs transfected with si-control or si-*LOX*. Bottom graph shows calculation results of the infiltration area. Scale bar 200 µm.

(E) LOX activity assay for recombinant active LOX protein by concentration-dependent manner (0.04, 0.102, 0.256, 0.64, 1.6, 4μg/mL).

(F) LOX activity assay for BAPN-treated recombinant active LOX protein.

(G) Infiltration of GBM cells into remodeled ECM treated with rhLOX and schematic model of the experimental procedure. Scale bar 1 mm.

(H) Invasion of GBM cells after pre-incubation in DMEM medium treated with rhLOX in a concentration dependent manner of 6mg/mL collagen coated transwell. Scale bar 200 µm.

(I) H&E staining image and number of X01 GBM cells that infiltrated into collagen-based matrix pre-incubated with rhLOX in a concentration-dependent manner. Scale bar, 200 µm.

**P* < 0.05, ***P* < 0.01, ****P* < 0.001, *****P* < 0.0001.

**
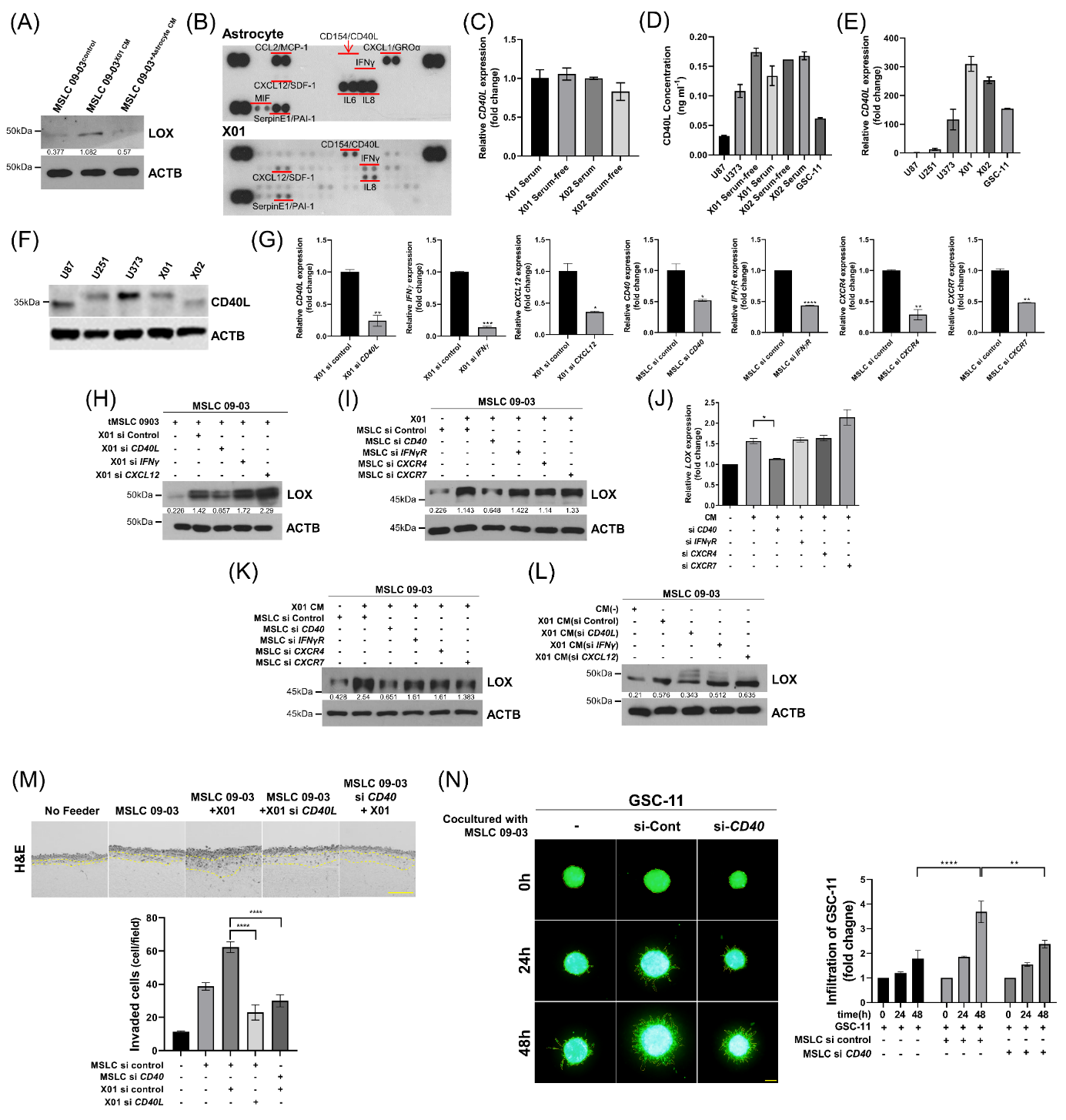
**

**Figure S3:** MSLCs reprogrammed by CD40L secreted by GBM cells via CD40 in MSLCs.

(A) Western blotting using MSLC treatment control CM (MSLC culture media), X01 CM, or astrocyte CM.

(B) Whole blot of cytokine array in each astrocyte and X01 cultured medium of X01 cells.

(C) qRT-PCR of *CD40L* expression by serum in indicated GBM cells.

(D) Enzyme-linked immunosorbent assay for CD40L levels in indicated GBM cells (n=2).

(E) qRT-PCR of *CD40L* expression in indicated GBM cells.

(F) Western blot of CD40L expression in indicated GBM cells.

(G) Transfection efficiency for each siRNA confirmed by qRT-PCR.

(H) Western blot for LOX in MSLCs co-cultured with X01 cells depleted of cytokine genes by siRNA.

(I) Western blot of LOX expression in MSLCs with each cytokine receptor gene knockdown co-cultured with X01 cells.

(J) RT-qPCR of *LOX* expression in MSLCs with each cytokine receptor gene knockdown treated with X01 CM.

(K) Western blot of LOX expression in MSLCs with each cytokine receptor gene knockdown treated with X01 CM.

(L) Western blot of LOX expression in MSLCs treated with CM of X01 depleted of cytokine genes.

(M) H&E staining image and number of X01 GBM cells that infiltrated into collagen-based matrix in main figure (**G**). Scale bar, 200 µm.

(N) GSC-11 spheroid cells infiltration with 3D collagen-based matrix co-cultured with MSLCs transfected with si-control or si-*CD40*. Right graph shows calculation results of the infiltration area. Scale bar 200 µm.

**P* < 0.05, ***P* < 0.01, ****P* < 0.001, *****P* < 0.0001.


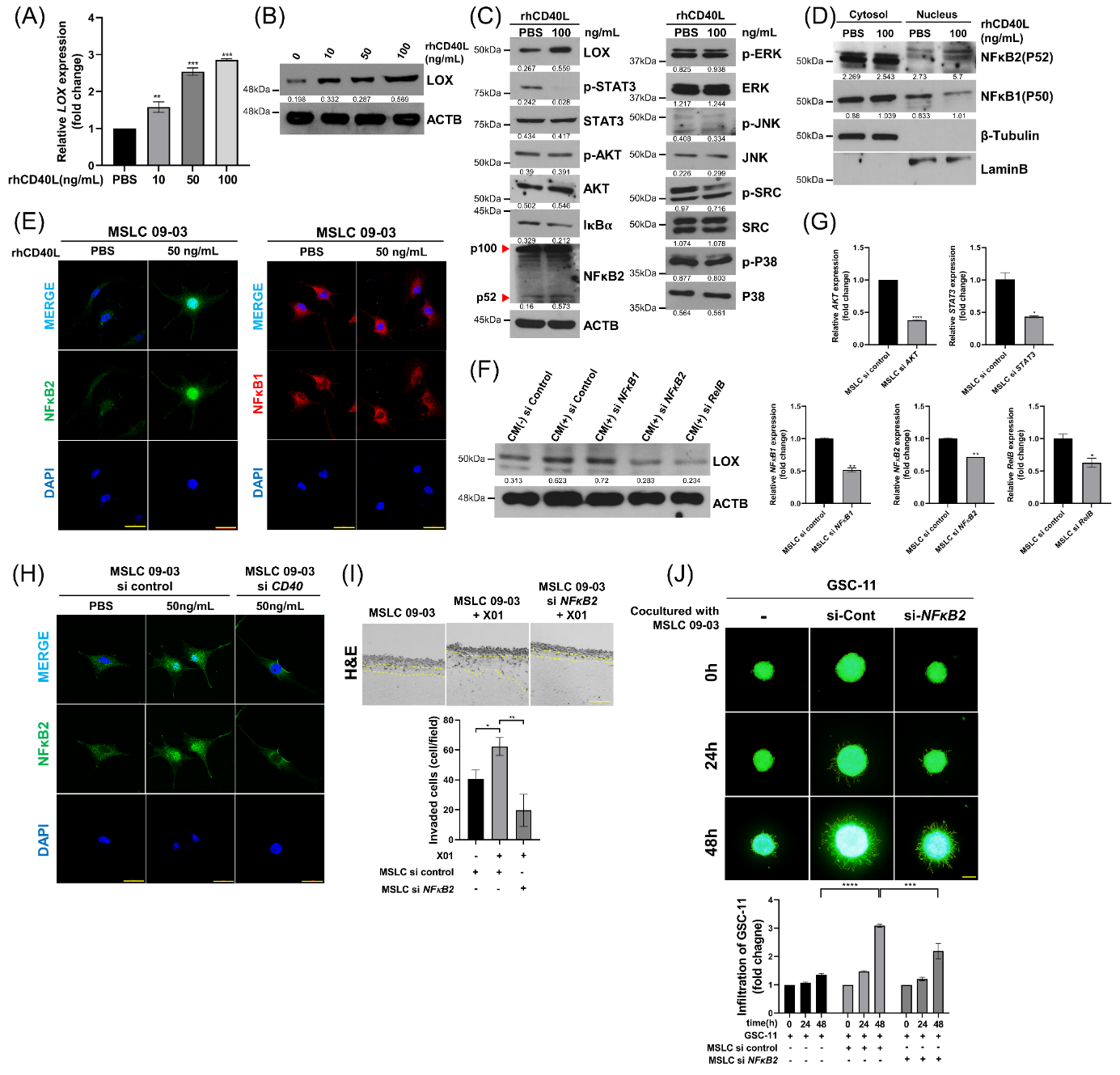


**Fig. S4.** CD40L promotes LOX expression through activation of NFkB2 by facilitating CD40 signaling pathway.

(A-B) qRT-PCR and western blot of *LOX* expression levels in MSLCs treated with rhCD40L in a dose-dependent manner.

(C) Western blot in MSLCs CD40 downstream effector activation status through PBS or 100ng/mL recombinant human CD40L (rhCD40L).

(D) Western blot of the activation of NFκB1 or NFκB2 by rhCD40L through cytosol-nucleus fraction in MSLCs.

(E) Immunocytochemistry (ICC) in MSLCs and nucleus translocation of NFκB1 or NFκB2 by rhCD40L. Scale bar 50µm.

(F) Western blot of LOX expression in MSLCs treated with X01 CM and transfected with indicated siRNA.

(G) Transfection efficiency for each siRNA confirmed by qRT-PCR.

(H) NFκB2 nucleus translocation in MSLC si-control or MSLC si-*CD40* in treated with rhCD40L. Scale bar 50µm.

(I) H&E staining image and number of X01 cells that infiltrated into the collagen-based matrix in main fig. 4J. Scale bar, 200 µm.

(J) GSC-11 spheroid cells infiltration with 3D collagen-based matrix co-cultured with MSLCs transfected with si-control or si-*NFκB2*. Bottom graph shows calculation results of the infiltration area. Scale bar 200µm.

**P* < 0.05, ***P* < 0.01, ****P* < 0.001, *****P* < 0.0001.

**
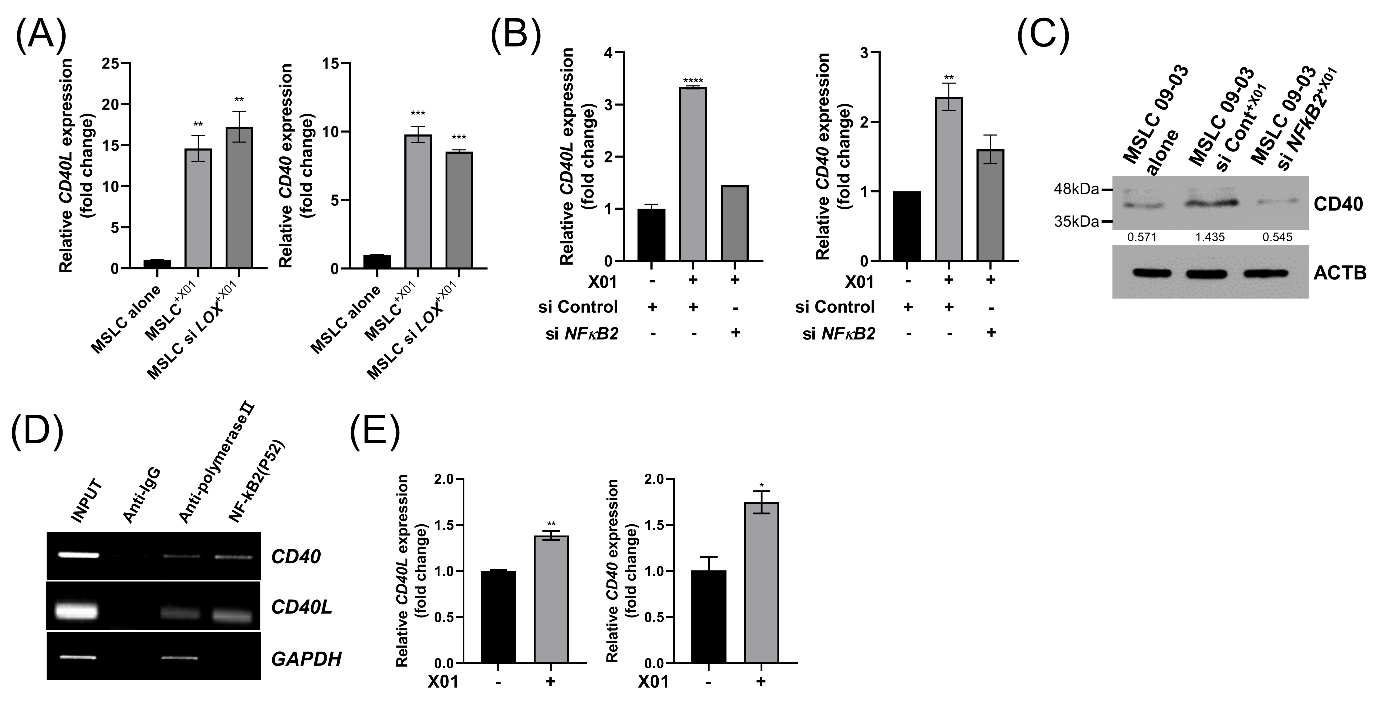
**

**Fig. S5.** CD40L induces feedback loop in MSLCs.

(A) qRT-PCR of expression of *CD40L* and *CD40* in *LOX* knockdowned MSLCs.

(B-C) qRT-PCR and Western blot of *CD40L* and *CD40* expression levels in *NFκB2* knockdown MSLCs.

(D) Chromatin immunoprecipitation (ChIP) assay for NFκB2 binding to CD40L and CD40 promotor.

(E) qRT-PCR of *CD40L* and *CD40* expression in BM-MSCs after co-cultured with X01.

(F) qRT-PCR of relative mRNA expression of *CD40L* and *CD40* in X01 cells and MSLCs.

**P* < 0.05, ***P* < 0.01, ****P* < 0.001, *****P* < 0.0001.

**
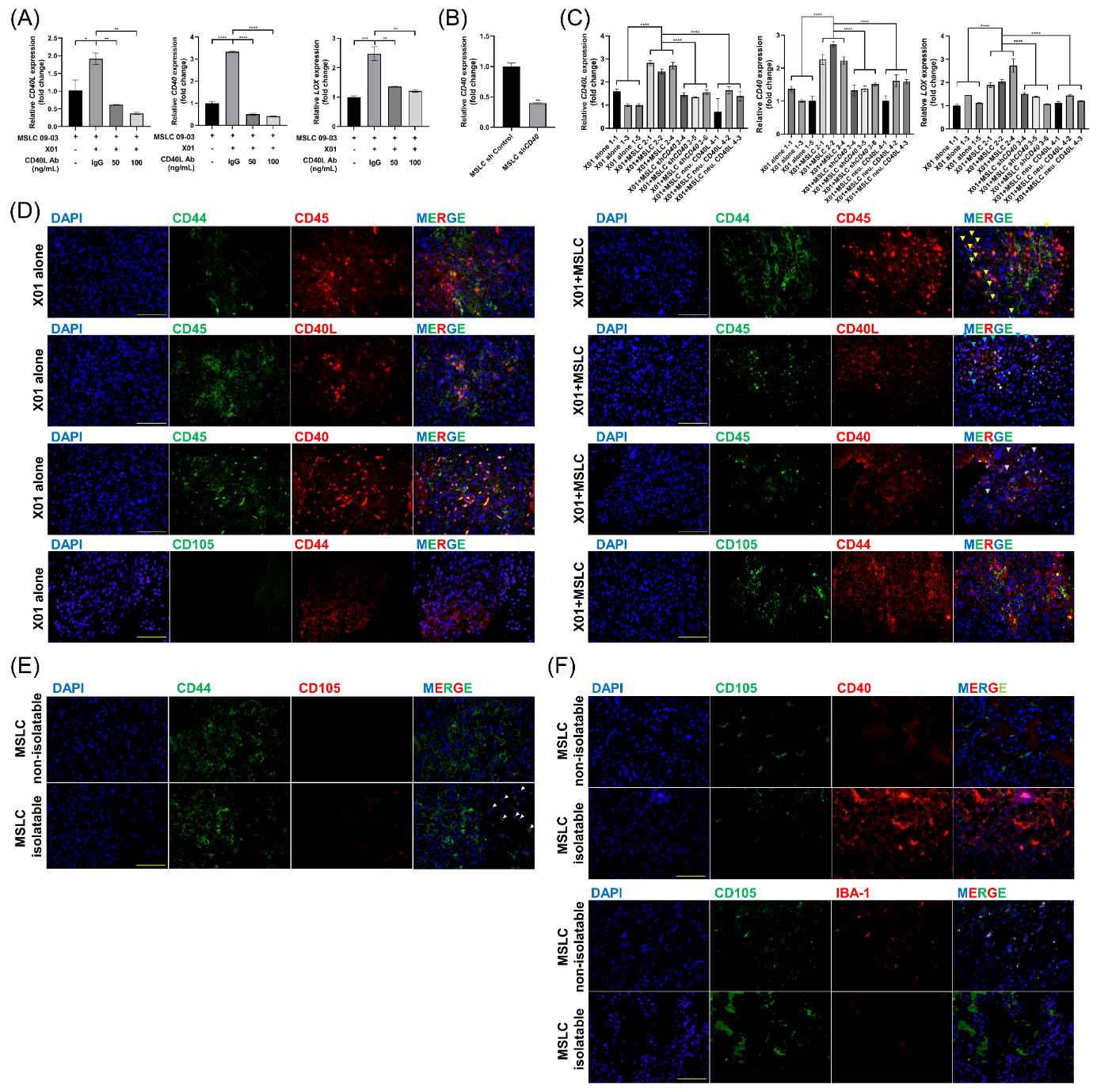
**

**Figure S6:** Effects of CD40L-neutralizing antibody treatment in vivo and in vitro.

(A) qRT-PCR of LOX expression levels in MSLCs co-cultured with X01 cells treated with CD40 Ab in a dose-dependent manner.

(B) Efficiency for CD40 shRNA confirmed by qRT-PCR in MSLCs.

(C) Detailed expression levels of CD40L, CD40, and LOX for Figure 5D. (n=3 mouse/group).

(D) IHC of GBM marker (CD105^-^, CD44^+^, CD45^+^), MSLC marker (CD105^+^, CD44^+^, CD45^-^) in X01 alone and X01+MSLC co-injection mouse tissue sample. And expression of CD40 and CD40L in each cell (X01 and MSLC). Yellow inverse triangle present CD44^+^CD45^-^ cells, light blue inverse triangle present CD45^-^CD40L^+^ cells and white inverse triangle present CD45^-^CD40^+^ cells. Scale bar, 100 µm.

(E) IHC of GBM, MSLC marker (CD44), MSLC marker (CD105) in MSLC-isolatable and MSLC-non-isolatable patient samples. Scale bar, 100 µm.

(F) IHC of co-stained CD105, CD40 and CD105, IBA-1 in MSLC-isolatable and MSLC-non-isolatable patient samples. Scale bar, 100 µm.

**P* < 0.05, ***P* < 0.01, ****P* < 0.001, *****P* < 0.0001.


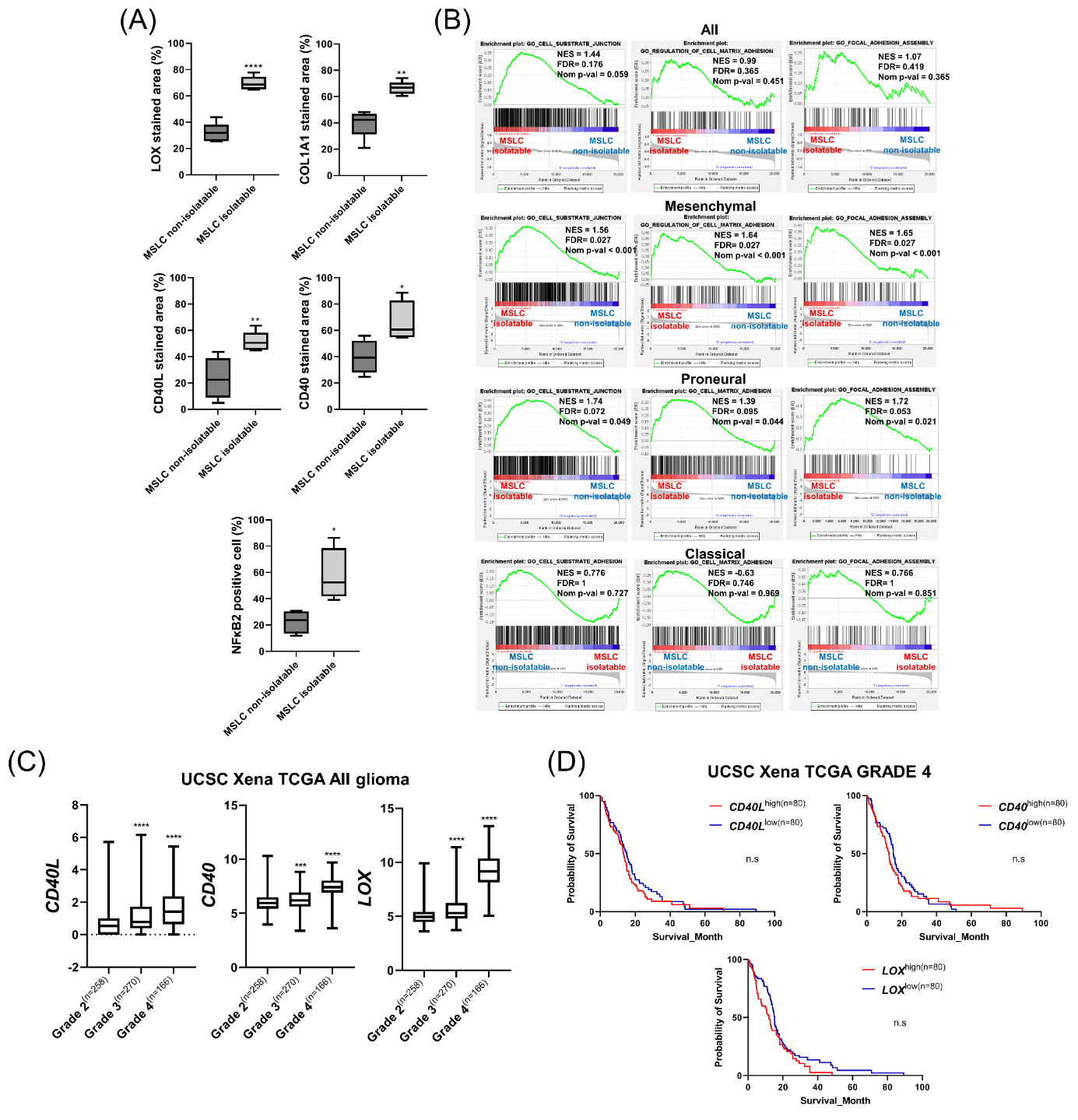


**Figure S7:** CD40L, CD40, LOX related clinical outcome.

(A) Semi-quantitative analysis of the IHC. LOX, COL1A1, CD40L, CD40 were calculated at the cytosol and extracellular matrix region except for nucleus, NFκB2 was calculated at the nucleus.

(B) Detail of GSEA analysis in Figure 6C.

(C) Expression levels of *CD40L*, *CD40*, and *LOX* according to patient grade.

(D) Kaplan-Meier survival curves of Grade 4 patients (TCGA) with high or low indicated median expression of gene.

n.s : not significant, **P* < 0.05, ***P* < 0.01, ****P* < 0.001, *****P* < 0.0001.

**Table S1. Sequences of siRNAs**

| **Name** |  | **Sequence** | | |
| --- | --- | --- | --- | --- |
| si control | Sence | | 5’-CCUCGUGCCGUUCCAUCAGGUAGUU-3’ |  |
|  | Antisence | | 5’-CUACCUGAUGGAACGGCACGAGGUU-3’ |  |
| si *LOX* | Sence | | 5’-CUACUACGAUACUUAUGAAUU-3’ |  |
|  | Antisence | | 5’-UUCAUAAGUAUCGUAGUAGUU-3’ |  |
| si *CD40L* | Sence | | 5’-GGAUAUAAUGUUAAACAAAUU-3’ |  |
|  | Antisence | | 5’-UUUGUUUAACAUUAUAUCCUU-3’ |  |
| si *IFNγ* | Sence | | 5’-AGAUGUAGCGGAUAAUGGAUU-3’ |  |
|  | Antisence | | 5’-UCCAUUAUCCGCUACAUCUUU-3’ |  |
| si *CXCL12* | Sence | | 5’-GCAUUGACCCGAAGCUAAAUU-3’ |  |
|  | Antisence | | 5’-UUUAGCUUCGGGUCAAUGCUU-3’ |  |
| si *CD40* | Sence | | 5’-GGAUAUAUGUAUAAAUACAUU-3’ |  |
|  | Antisence | | 5’-UGUAUUUAUACAUAUAUCCUU-3’ |  |
| si *IFNγR* | Sence | | 5’-CAAUAUUUCUCAUCAUUAUUU-3’ |  |
|  | Antisence | | 5’-AUAAUGAUGAGAAAUAUUGUU-3’ |  |
| si *CXCR4* | Sence | | 5’-CCAUCUACUCCAUCAUCUUUU-3’ |  |
|  | Antisence | | 5’-AAGAUGAUGGAGUAGAUGGUU-3’ |  |
| si *CXCR7* | Sence | | 5’-CUGAUGAAGGCCUUCAUCUUU-3’ |  |
|  | Antisence | | 5’-AGAUGAAGGCCUUCAUCAGUU-3’ |  |
| si *AKT* | Sence | | 5’-GCACCUUCAUUGGCUACAAUU-3’ |  |
|  | Antisence | | 5’-UUGUAGCCAAUGAAGGUGCUU-3’ |  |
| si *STAT3* | Sence | | 5’-CACAAUCUACGAAGAAUCAUU-3’ |  |
|  | Antisence | | 5’-UGAUUCUUCGUAGAUUGUGUU-3’ |  |
| si *NFκB1* | Sence | | 5’-CCAUAUGAGCCAGAGUUUAUU-3’ |  |
|  | Antisence | | 5’-UAAACUCUGGCUCAUAUGGUU-3’ |  |
| si *NFκB2* | Sence | | 5’-GUGUCAUUGAGCAGAUAGUUU-3’ |  |
|  | Antisence | | 5’-ACUAUCUGCUCAAUGACACUU-3’ |  |
| si *RelB* | Sence | | 5’-CCAUUGAGCGGAAGAUUCAUU-3’ |  |
|  | Antisence | | 5’-UGAAUCUUCCGCUCAAUGGUU-3’ |  |

**Table S2. List of primers used in qRT-PCR**

| Name | Forward primer (5’-3’) | Reverse primer (5’-3’) |
| --- | --- | --- |
| *GAPDH* | GGCAAATTCCATGGCACCGTCAAGG | GCCAGCATCGCCCCACTTGATTTTG |
| *ACTB* | CATCCGCAAAGACCTGTACG | CCTGCTTGCTGATCCACATC |
| *ACAN* | CCTCTGGACAACCAGGTGTT | AAACCAGGTCAGGGACTCCT |
| *BCAN* | CAGCCCAGGAGAAGTCACTC | GGGATGCTAGGTTCCTCTCC |
| *NCAN* | CACGTCACAACATGGAGACC | TCTTCTTCCCCACTGGACAC |
| *VCAN* | GAGGAGGCTGCAAAAGAGTG | TACAGGGTTCTCACCCCAAG |
| *FN* | ACCAACCTACGGATGACTCG | GCTCATCATCTGGCCATTTT |
| *OPN* | AGACCTGACATCCAGTACCCTG | GTGGGTTTCAGCACTCTGGT |
| *COL1A1* | AATCCATCGGTCATGCTCTC | CCTGGATGCCATCAAAGTCT |
| *COL1A2* | ACGGTGATAAAGGTCATGCT | TTTTCCACCTTGAACACCCT |
| *COL3A1* | TGCCCCGTATTATGGAGATG | TAGCGGGGTTTTTACGAGAA |
| *COL4A1* | AGCCTGGTGAGTTTTATTTCG | CGCTCTCCTTTCAATCCTACA |
| *COL6A1* | CGAATGCGAGATTTTGGACA | AAGTCCTTGGCAATCTCGAA |
| *LOX* | CGGCGGAGGAAAACTGTCT | TCGGCTGGGTAAGAAATCTG |
| *LOXL1* | AGAGCCTCTCTGTCCACCAG | GTACACCTGCCCGTTGTTCTTCT |
| *LOXL2* | CCTGGGGAGAGGACATACAA | CTCGCAGGTGACATTCTTCA |
| *LOXL3* | CAACGCGGCCTTCTACAG | GGTGTCATTGGCACGATAGA |
| *LOXL4* | CGACAGCCACTACTACAGGAAA | CTGGTGGATCCAGAAGGAGTT |
| *MMP1* | CAGCGACTCTAGAAACACAAG | GGTTTCAGCATCTGGTTTCC |
| *MMP2* | TTGACGGTAAGGACGGACTC | ACTTGCAGTACTCCCCATCG |
| *MMP9* | ACCACCACAACATCACCTAT | CACCAAACTGGATGACGATG |
| *MMP13* | TTGAGCTGGACTCATTGTCG | GGAGCCTCTCAGTCATGGAG |
| *CD40L* | ATGGGAAACAGCTGACCGTT | GATTGTTGCCCGCAAGGTTT |
| *CD40* | ATGCTGCTGAATGATGGGTA | CTCCCTTTAACCAACCCTCC |
| *IFNγ* | GTGGAGACCATCAAGGAAGACA | CGCTTCCCTGTTTTAGCTGC |
| *CXCL12* | GCCGATTCTTCGAAAGCCAT | TTTGGCTGTTGTGCTTACTTG |
| *IFNγR* | GACGGAAGTGACGTAAGGC | TAGTTGGTGTAGGCACTGAGG |
| *CXCR4* | CACTATGGGAAAAGATGGGGA | TCTGCAAAAGAGGCAAAGGA |
| *CXCR7* | TTCTCCTACGTGGTGGTCTT | AAGCTGTAGAGGACAGGGTT |
| *AKT* | TCAAAGAAGTCAAAGGGGCT | CAGGTCTTGATGTACTCCCC |
| *STAT3* | CCCCCTCAAAAGTCATCCTGC | GGAGTCCAGTACACGGTGAG |
| *NFκB1* | GCAGTGCCATCTGTGGTTG | GCGCCGCTTAGGAGGG |
| *NFκB2* | GGAGGGCCTTTAGCGGAC | GCCCGGCTCTGTCTAGTG |
| *RelB* | GTTTCCAGGAGCACAGATGA | AGGGTGACCGTGCTCAG |

**Table S3. List of primers used in ChIP-assay**

| Name | Forward primer (5’-3’) | Reverse primer (5’-3’) |
| --- | --- | --- |
| *GAPDH* | TACTAGCGGTTTTACGGGCG | TCGAACAGGAGGAGCAGAGAGCGA |
| ChIP-*LOX*-1 | GTCCTTCCACTATGCAACTG | GTGGTGGACCTCTGCTGAT |
| ChIP-*LOX*-2 | GACATGTGCCACCATGCC | CGGGCGGATCATTATCATTC |
| ChIP-*CD40L*-1 | GTACCTAAAGCTCCCAGCC | GAAAGTGTGCTGCCACCAAG |
| ChIP-*CD40L*-2 | GGGTAAGTCCTACATGCTCAC | CTTTGGAGATGCAGGCAAATTC |
| ChIP-*CD40*-1 | CAAGATGCGTCCCTAAACTCC | GGCGGGGTCTTCAAAGACC |
| ChIP-*CD40*-2 | GGATGCATGGATGAATGGATG | GGGAGGCGTTTCAAGGAAGA |

**Table S4. Reaction cycle of PCR**

**a) ChIP-assay reaction cycle, b) qRT-PCR reation cycle**

**a.**

| Step | Degree | Time |
| --- | --- | --- |
| Initial Denaturation | 95 ºC | 3 min |
| Denaturation* | 95 ºC | 20 sec |
| Annealing* | 60 ºC | 30 sec |
| Extension* | 72 ºC | 30 sec |
| Final Extension | 72 ºC | 2 min |

*** Repeat for a total 32 cycles**

**b.**

| Step | Degree | Time |
| --- | --- | --- |
| Initial Denaturation | 95 ºC | 5 min |
| Denaturation* | 95 ºC | 5 sec |
| Annealing* | 60 ºC | 10 sec |
| Extension* | 72 ºC | 10 sec |
| Melting | 65 ºC to 95ºC |  |

*** Repeat for a total 40 cycles**

**Table S5. The cohorts of MSLC Isolatable and non-Isolatable patients for IHC stained tissue used in this study**

| **tMSLC** | **Patient No.** | **MGMT** | **Gender** | **IDH-1 Mutation** | **EGFR amplification** | **LOH1p** | **LOH19q** | **LOH1p19q** |
| --- | --- | --- | --- | --- | --- | --- | --- | --- |
| tMSLC Isolatable | Patient 1 | unmethyl | M | wt | Amplification | intact | intact | nonCodel |
|  | Patient 2 | unmethyl | M | wt | Amplification | intact | intact | nonCodel |
|  | Patient 3 | unmethyl | M | wt | Amplification | intact | intact | nonCodel |
|  | Patient 4 | unmethyl | M | wt | Amplification | intact | intact | nonCodel |
|  | Patient 5 | methyl | F | wt | Amplification | intact | intact | nonCodel |
|  | Patient 6 | unmethyl | M | wt | wt | intact | intact | nonCodel |
|  | Patient 7 | unmethyl | M | wt | Amplification | LOH | LOH | Codel |
|  | Patient 8 | unmethyl | M | wt | wt | intact | intact | nonCodel |
| tMSLC non-Isolatable | Patient 9 | unmethyl | F | wt | Amplification | LOH | LOH | Codel |
|  | Patient 10 | unmethyl | F | wt | Amplification | intact | intact | nonCodel |
|  | Patient 11 | unmethyl | F | wt | Amplification | intact | intact | nonCodel |
|  | Patient 12 | unmethyl | F | wt | Amplification | intact | intact | nonCodel |
|  | Patient 13 | unmethyl | F | wt | Amplification | intact | intact | nonCodel |
|  | Patient 14 | methyl | F | wt | Amplification | intact | intact | nonCodel |
|  | Patient 15 | unmethyl | F | wt | Amplification | LOH | LOH | Codel |
| wt : wild type, methyl : methylation, unmethyl : unmethylation, LOH : loss of heterozygosity, Codel : codeletion, nonCodel : non-codeletion | | | | | | | | |

**Table S6. The cohorts of MSLC Isolatable and non-isolatable patients for GSEA analysis**

| **Clinical factor** |  | **MSLC isolatable** | **MSLC non-isolatable** |
| --- | --- | --- | --- |
| **Molecular subtype** | **Mesenchymal** | **5/19** | **3/12** |
|  | **proneural/neural** | **8/19** | **6/12** |
|  | **classical** | **6/19** | **3/12** |
| **Gender** | **M** | **14/19** | **6/12** |
|  | **F** | **5/19** | **6/12** |
| **IDH-1 muation** | **wt** | **17/19** | **12/12** |
|  | **mut** | **2/19** | **0/12** |
| **loh1p** | **intact** | **16/19** | **9/12** |
|  | **LOH** | **3/19** | **2/12** |
|  | **n.a** | **0/19** | **1/12** |
| **loh19q** | **intact** | **15/19** | **8/12** |
|  | **LOH** | **4/19** | **3/12** |
|  | **n.a** | **0/19** | **1/12** |
| **1p19q** | **nonCodel** | **16/19** | **11/12** |
|  | **Codel** | **3/19** | **0/12** |
|  | **n.a** | **0/19** | **1/12** |
| **MGMT** | **unmethylation** | **12/19** | **7/12** |
|  | **methylation** | **7/19** | **5/12** |
| n.a : not detected wt : wild type, LOH : loss of heterozygosity, Codel : codeletion, nonCodel : non-codeletion | | | |
