# Supplementary figures and images for: Glioblastoma‐educated mesenchymal stem‐like cells promote glioblastoma infiltration via extracellular matrix remodelling in the tumour microenvironment
Source: Clin Transl Med. 2022 Jul 31;12(8):e997. doi: 10.1002/ctm2.997 (PMC9339241; doi:10.1002/ctm2.997)

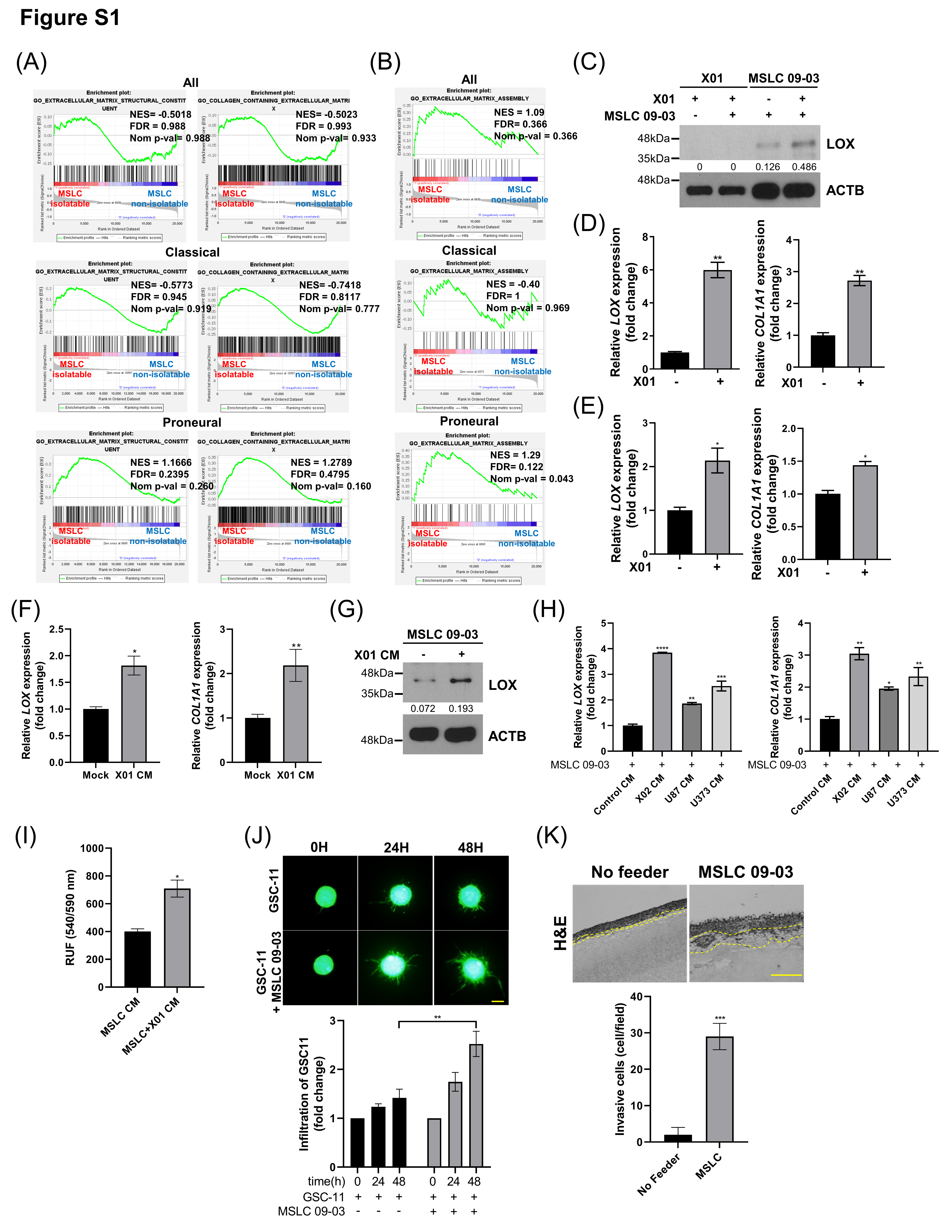

Supplement: Supplementary file 2 — ctm2997‐sup‐0002‐FigureS1.tif [file CTM2-12-e997-s006.tif]

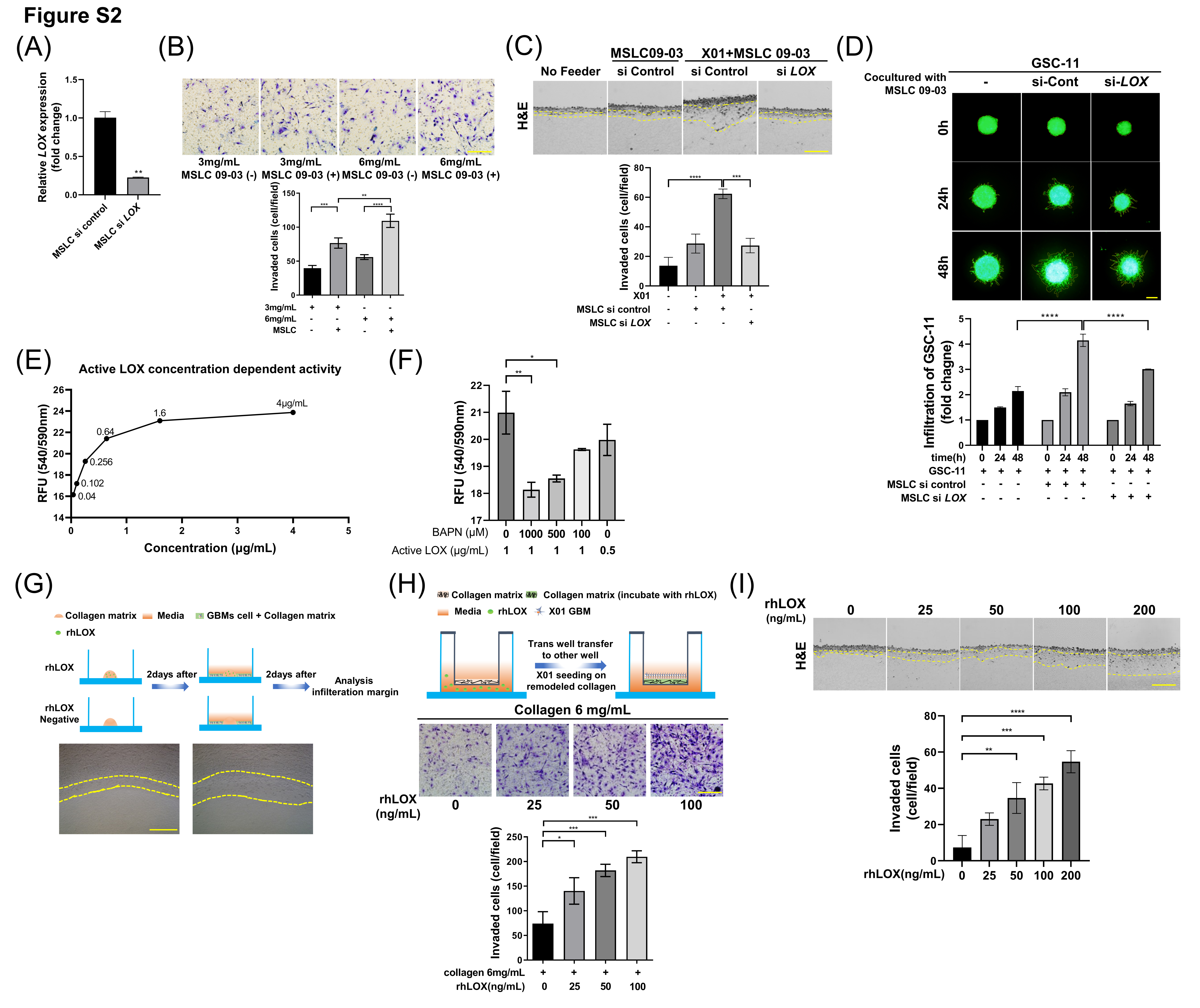

Supplement: Supplementary file 3 — ctm2997‐sup‐0003‐FigureS2.tif [file CTM2-12-e997-s005.tif]

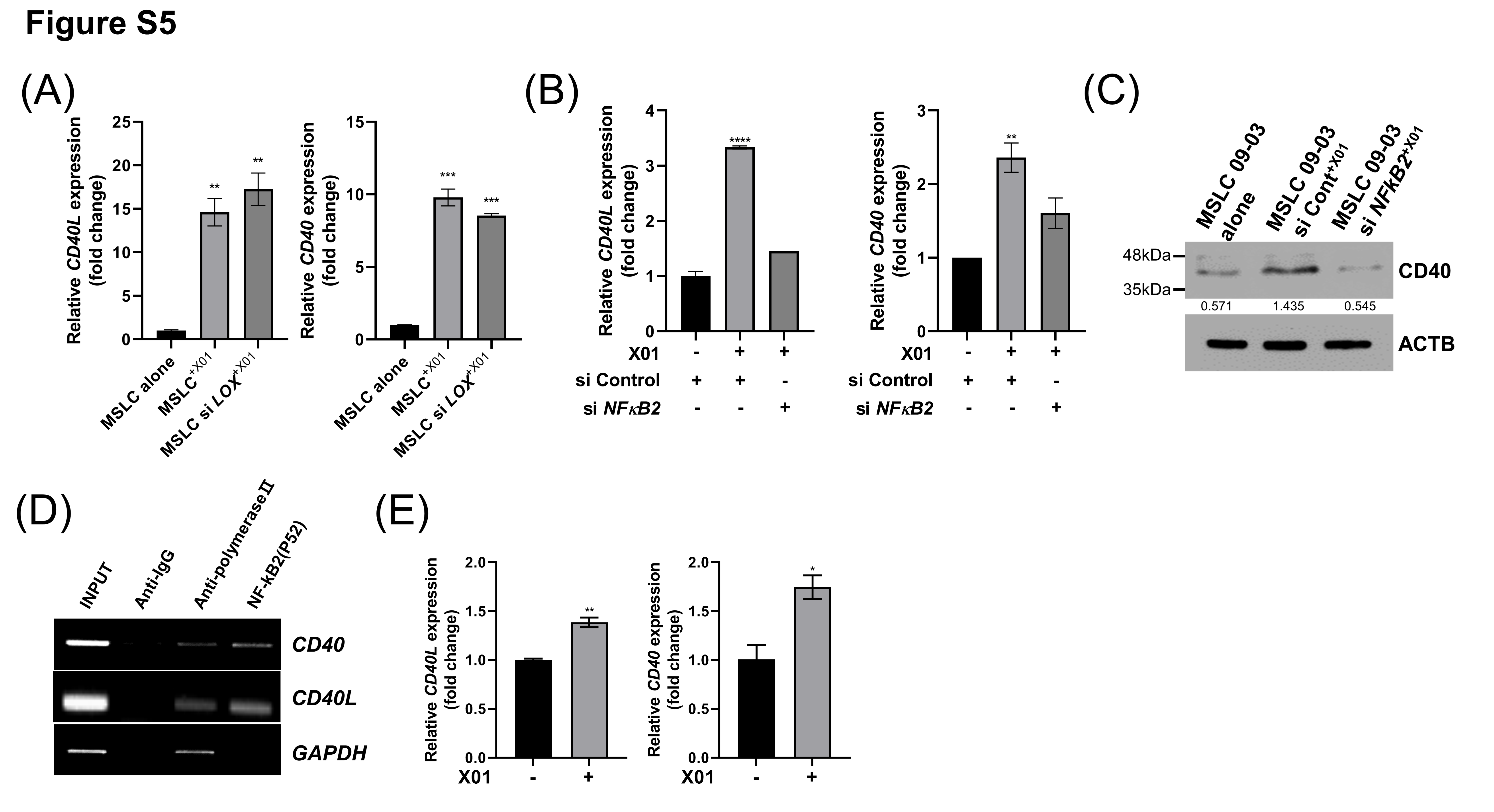

Supplement: Supplementary file 6 — ctm2997‐sup‐0006‐FigureS5.tif [file CTM2-12-e997-s007.tif]

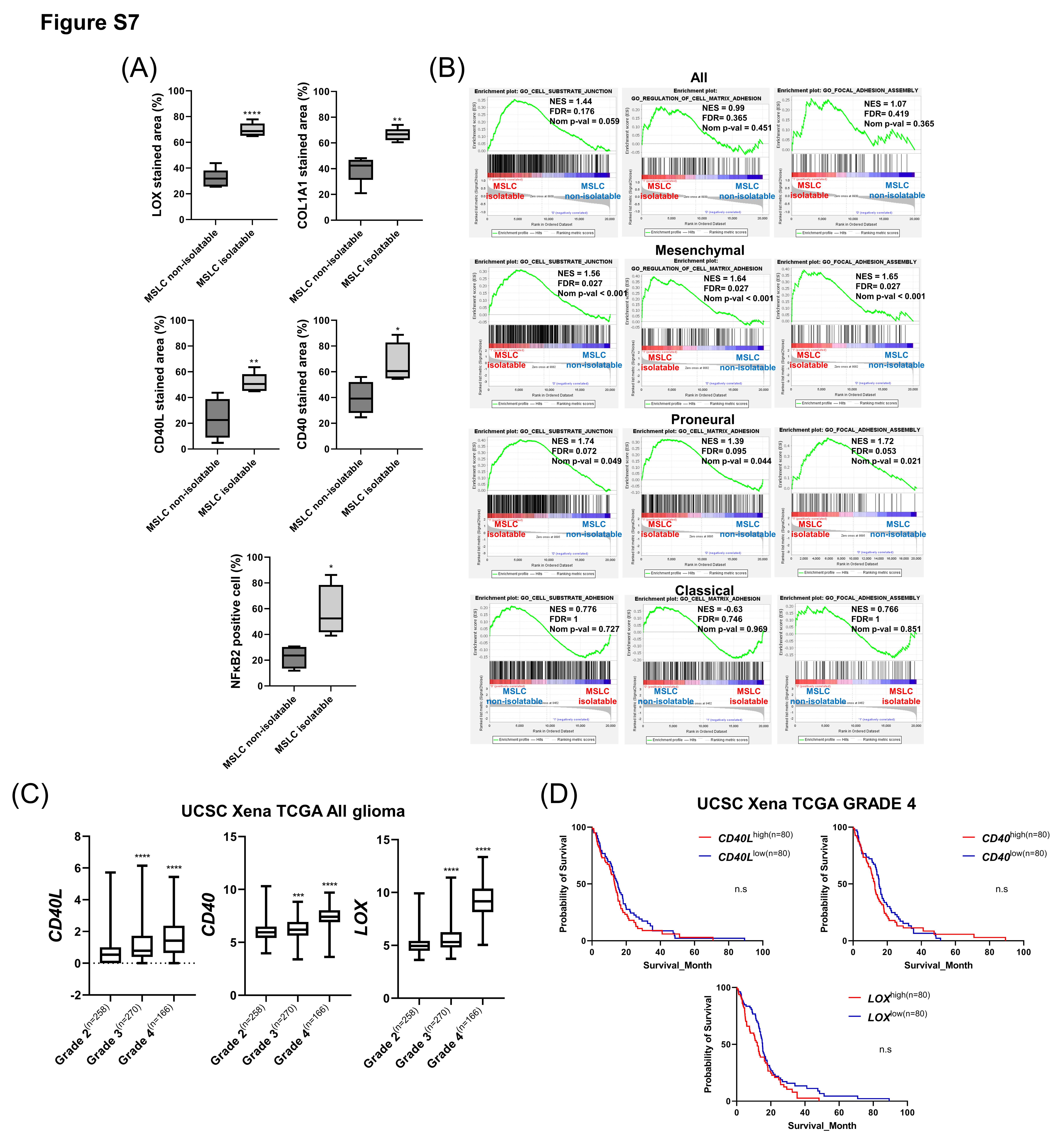

Supplement: Supplementary file 8 — ctm2997‐sup‐0008‐FigureS7.tif [file CTM2-12-e997-s002.tif]
